# Comparison of Low‐Rank Denoising Methods for Dynamic Deuterium MRSI at 7 T

**DOI:** 10.1002/nbm.70125

**Published:** 2025-08-31

**Authors:** Anna Duguid, Fabian Niess, Wolfgang Bogner, Lukas Hingerl, Viola Bader, Sabina Frese, Aaron Osburg, Bernard Lanz, Brayan Alves, Cristina Cudalbu, Simon Daniel Robinson, Korbinian Eckstein, Bernhard Strasser

**Affiliations:** ^1^ High Field MR Center, Department for Biomedical Imaging and Image‐Guided Therapy Medical University of Vienna Vienna Austria; ^2^ Christian Doppler Laboratory for MR Imaging Biomarkers (BIOMAK), Department for Biomedical Imaging and Image‐Guided Therapy Medical University of Vienna Vienna Austria; ^3^ CIBM Center for Biomedical Imaging Lausanne Switzerland; ^4^ Animal Imaging and Technology, EPFL Lausanne Switzerland

**Keywords:** 3D MRSI, denoising, deuterium, low‐rank, MRS, whole‐brain metabolite mapping

## Abstract

Dynamic deuterium (^2^H)‐MRSI enables mapping of metabolic fluxes in vivo, but its sensitivity is hampered by the low ^2^H gyromagnetic ratio and ^2^H‐labelled metabolite concentrations. Low‐rank denoising can enhance MRSI sensitivity by separating signal from noise. Several methods have been proposed, but the optimal approach for dynamic ^2^H‐MRSI remains unclear. This work compares six low‐rank denoising methods for dynamic ^2^H‐MRSI: four variations of spatiospectral partial separability (PS)—Global PS, Local PS, Stacked PS and SPectral and temporal INtegration for SVD (SPIN‐SVD)—as well as global–local higher order singular value decomposition (GL‐HOSVD) and tensor Marchenko–Pastur principal component analysis (tMPPCA). Performance was evaluated using realistic ^2^H‐MRSI brain simulations across a range of noise and *B*_0_ inhomogeneity levels, including a lesion model featuring focal metabolic alteration, and in vivo data. For simulations at the in vivo SNR and *B*_0_ level, the concentration root‐mean‐square errors (RMSE) of the fitted metabolite maps against gold standard maps were reduced by 29.3%, 24.4%, 20.2%, 33.4%, 23.5% and 21.9% following denoising with Global PS, Local PS, Stacked PS, SPIN‐SVD, GL‐HOSVD and tMPPCA, respectively. Across all simulated noise levels and all but the highest *B*_0_ inhomogeneity level, SPIN‐SVD achieved the lowest concentration RMSE and best preserved spatial distributions of ^2^H‐labelled water (HDO) and lactate (Lac), including lesion‐associated changes. While Global PS and Stacked PS reduced noise variance, they failed to preserve signal variations in simulated and in vivo data. SPIN‐SVD, GL‐HOSVD and tMPPCA reduced noise in vivo while maintaining spatial and temporal metabolite variations. GL‐HOSVD and tMPPCA performed similarly, with tMPPCA preferred for its computational efficiency. Both SPIN‐SVD and tMPPCA are suitable for denoising dynamic ^2^H‐MRSI, with SPIN‐SVD preferable for clinical applications owing to its simple implementation and superior preservation of local metabolic alterations, and tMPPCA better suited for absolute quantification of very low SNR metabolites.

## Graphical abstract

Six low‐rank denoising methods for dynamic ^2^H‐MRSI were compared using simulated and in vivo data: Global PS, Local PS, Stacked PS, SPIN‐SVD, GL‐HOSVD and tMPPCA. SPIN‐SVD performed best in simulations, while SPIN‐SVD, GL‐HOSVD and tMPPCA performed well in vivo. GL‐HOSVD and tMPPCA performed similarly, with tMPPCA preferred for computational efficiency.

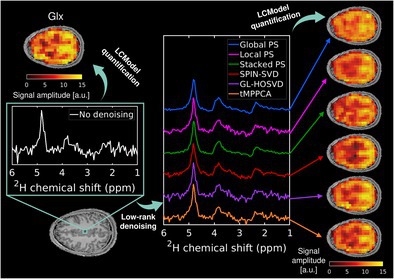

**Abbreviations**
^2^H: deuterium
5D: five dimensional
CSF: cerebrospinal fluid
FID: free induction decay
FOV: field of view
FWHM: full width at half maximum
Glc: glucose
GL‐HOSVD: global–local higher‐order singular value decomposition
Glx: glutamate + glutamine
GM: grey matter
HDO: deuterated water
HOSVD: higher‐order singular value decomposition
Lac: lactate
MPPCA: Marchenko–Pastur principal component analysis
MRSI: magnetic resonance spectroscopy imaging
PS: partial separability
RMSE: root‐mean‐square error
SNR: signal‐to‐noise ratio
SPIN‐SVD: SPectral and temporal INtegration for singular value decomposition
SVD: singular value decomposition
TI1: first inversion time
TI2: second inversion time
tMPPCA: tensor Marchenko–Pastur principal component analysis
WM: white matter

## 1 Introduction

Deuterium (^2^H) MRSI is an emerging method for mapping in vivo concentrations of ^2^H‐labelled metabolites, typically paired with the administration of ^2^H‐labelled substrates such as glucose, acetate, water or fumarate [1, 2, 3]. By measuring the concentrations of ^2^H‐labelled substrates and their downstream metabolites over time (dynamic ^2^H‐MRSI), fluxes through key metabolic pathways, such as glycolysis and the tricarboxylic acid cycle, could be inferred [4, 5, 6]. This technique holds promise for advancing our understanding of neurological diseases such as Alzheimer's disease, epilepsy and brain tumours, which are characterised by pathology‐driven alterations in brain glucose metabolism [7, 8, 9, 10, 11, 12]. Beyond research applications, ^2^H‐MRSI has significant potential for clinical translation, offering a non‐invasive approach for diagnosis, prognosis and monitoring of treatment response [5, 13, 14]. However, the sensitivity of ^2^H‐MRSI is limited by the ^2^H gyromagnetic ratio—which is 6.5 times lower than that of hydrogen [1]—and by the low concentrations of ^2^H‐labelled metabolites even following ^2^H‐labelled substrate administration [4, 15, 16, 17]. As a result, most ^2^H‐labelling studies to date have been conducted at steady state [1], as single‐voxel experiments [4, 5] or with low spatiotemporal resolution [6]. When whole‐brain data are acquired at high spatial and temporal resolution to capture metabolite spatial variations and kinetics, the signal‐to‐noise ratio (SNR) is low. Thus, techniques that preserve signal‐driven variations while effectively suppressing noise are highly desirable.

Low‐rank denoising methods aim to reduce the noise variance of MRSI data by exploiting low‐rank structures in the signal, such as spatiospectral partial separability (PS) or linear predictability [18]. These methods use a matrix factorisation technique—singular value decomposition (SVD)—to identify signal‐ and noise‐driven components in the data. More recently, low‐rank algorithms based on higher‐order SVD (HOSVD), a specific orthogonal form of Tucker decomposition, have been implemented for diffusion MRI and ^13^C‐MRSI [19, 20, 21, 22, 23, 24]. While SVD necessitates reshaping of multidimensional data into a matrix, leading to a partial loss of information in the data structure, HOSVD‐based methods retain the natural tensor structure of the data to utilise redundancy across all encoding dimensions [21].

Dynamic ^2^H‐MRSI is particularly well suited to low‐rank denoising. Unlike brain ^1^H‐MRSI spectra, which feature strong nuisance water and lipid signals, the signals of interest dominate brain ^2^H‐MRSI spectra. Additionally, the data are sparse in the spectral domain, with only four detectable spectral peaks in the brain: water (HDO), glucose (Glc), glutamate & glutamine (Glx) and lactate (Lac) [1]. This suggests that the spectra can be well approximated as a linear combination of a very small number of basis signals, making them well suited for denoising with low‐rank‐based methods. Furthermore, the concentrations of ^2^H‐labelled metabolites change slowly over time [15] and can be represented as a set of exponential functions with different decay or recovery time constants [25]. This additional redundancy in the temporal domain of dynamic compared with static MRSI data can be leveraged in low‐rank‐based approaches [25]. Despite this potential, the optimal denoising strategy for dynamic ^2^H‐MRSI data is still unclear. Moreover, although low‐rank denoising methods consistently increase the apparent spectral SNR of MRSI data, they can increase uncertainty in the metabolite concentration estimates in some cases [26]. This means robust evaluation of denoising approaches is essential.

This work compares six low‐rank denoising methods for dynamic 3D ^2^H‐MRSI following administration of [6,6’‐^2^H_2_]‐glucose. The first four methods, which are variations of spatiospectral PS—Global PS, Local PS, Stacked PS and SPectral and temporal INtegration for SVD (SPIN‐SVD)—reshape the data into a matrix before denoising using a low‐rank approximation. While Global PS and Local PS are established approaches, Stacked PS and SPIN‐SVD are novel PS variations introduced in this study. The other two methods, global–local HOSVD (GL‐HOSVD) and tensor Marchenko–Pastur principal component analysis (tMPPCA), are HOSVD‐based. The denoising methods are evaluated using realistic simulations of dynamic ^2^H‐MRSI brain data, including a lesion model with locally altered metabolism, and assessed for in vivo dynamic ^2^H‐MRSI of the human brain.

## 2 Theory

### 2.1 Low‐Rank Denoising

A measured noise‐perturbed matrix $D\in {\mathbb{C}}^{N\times M}$ can be expressed as:

(1) $$D=D_{0}+\varepsilon$$

where $D_{0}$ denotes the noiseless signal and ε denotes the measurement noise. Low‐rank denoising aims to reduce ε without perturbing $D_{0}$. The key assumption is that $D_{0}$ exhibits low‐rank structure; that is, it can be well‐approximated as a linear combination of only a small number, L, of components, where $L\ll \min\left(N,M\right)$.

According to the Eckart–Young–Minsky theorem, the best rank‐L approximation of D is given by its hard‐thresholded SVD:

(2) $$\hat{U}\hat{\Sigma }{\hat{V}}^{T}=\sum_{i=1}^{L}\sigma_{i}u_{i}v_{i}^{T},$$

where $\hat{U}\in {\mathbb{C}}^{N\times L}$ and $\hat{V}\in {\mathbb{C}}^{M\times L}$ contain left and right singular vectors $u_{i}$ and $v_{i}$, respectively, and ${\hat{V}}^{T}$ is the conjugate transpose of $\hat{V}$. $\hat{\Sigma }\in {\mathbb{R}}^{L\times L}$ is a diagonal matrix containing singular values $\sigma_{1}\geq \sigma_{2}\geq \ldots \geq \sigma_{L}\geq 0$. Use of Equation (2) for denoising assumes that for the SVD of D, all $\sigma_{j}<\sigma_{L}$ correspond to predominantly noise‐driven components. The hard‐thresholding approach of Equation (2) sets all $\sigma_{j}<\sigma_{L}$ to zero. An alternative approach to denoising, known as soft‐thresholding, shrinks the singular values toward zero by subtracting a constant threshold [26, 27], that is, $\tilde{\sigma_{j}}=\max\left(\sigma_{j}-\sigma_{L+1},0\right)$, where $\tilde{\sigma_{j}}$ is the soft‐thresholded jth singular value.

While SVD is a matrix operation, dynamic ^2^H‐MRSI data take the form of higher‐dimensional tensors, comprising two to three spatial dimensions, a spectral dimension and a temporal dimension. Two approaches to low‐rank denoising of such data are (1) unfolding the data into a matrix—known as ‘matricisation’ methods and (2) preserving (to some degree) the original tensor structure and using HOSVD.

The HOSVD of a K‐mode tensor, A, can be expressed as:

(3) $$A=S\times_{1}U_{1}\times_{2}U_{2}\ldots \times_{K}U_{K},$$

where $\times_{k}$ is the k‐mode tensor product and the $U_{k}$ are unitary matrices. The $U_{k}$ are left singular vector matrices computed through SVD of $A_{k}$, the k‐mode flattening of A. The flattening is obtained by unfolding the tensor along its kth dimension to form a matrix with columns corresponding to the kth dimension and all other dimensions concatenated along the rows.

S, known as the core tensor, is an orthogonal $\left(I_{1}\times I_{2}\times \ldots \times I_{K}\right)$‐tensor consisting of the transform coefficients. It is computed from multilinear multiplication of A with the conjugate transposes of the $U_{k}$:

(4) $$S=A\times_{1}U_{1}^{H}\times_{2}U_{2}^{H}\ldots \times_{K}U_{K}^{H},$$

Denoising can be achieved by thresholding the coefficients of S, analogous to thresholding the singular values of Σ in matrix decomposition.

### 2.2 Spatiospectral Partial Separability

MRSI data exhibit a low‐rank structure according to the spatiospectral PS model [18, 28]. The spatiospectral function $\rho \left(r,f\right)$ of static MRSI data can be expressed as:

(5) $$\rho \left(r,f\right)=\sum_{i=1}^{L}c_{i}\left(r\right)\varphi_{i}\left(f\right),$$

where $c_{i}$ and $\varphi_{i}$ are the spatial and spectral modes, respectively, r is the spatial location, f is the spectral frequency and L is the model order (i.e., the data are Lth‐order partially separable). The spatiospectral PS model assumes that Equation (5) holds for small values of L, reflecting the limited number of detectable resonances in MRSI data. In other words, at any given location, the spectrum can be well approximated as the weighted sum of a small set of spectral components, ${\left\{\varphi_{i}\left(f\right)\right\}}_{i=1}^{L}$, with the weights determined by the values of the corresponding spatial components, ${\left\{c_{i}\left(r\right)\right\}}_{i=1}^{L}$, at that location.

**FIGURE 1:**
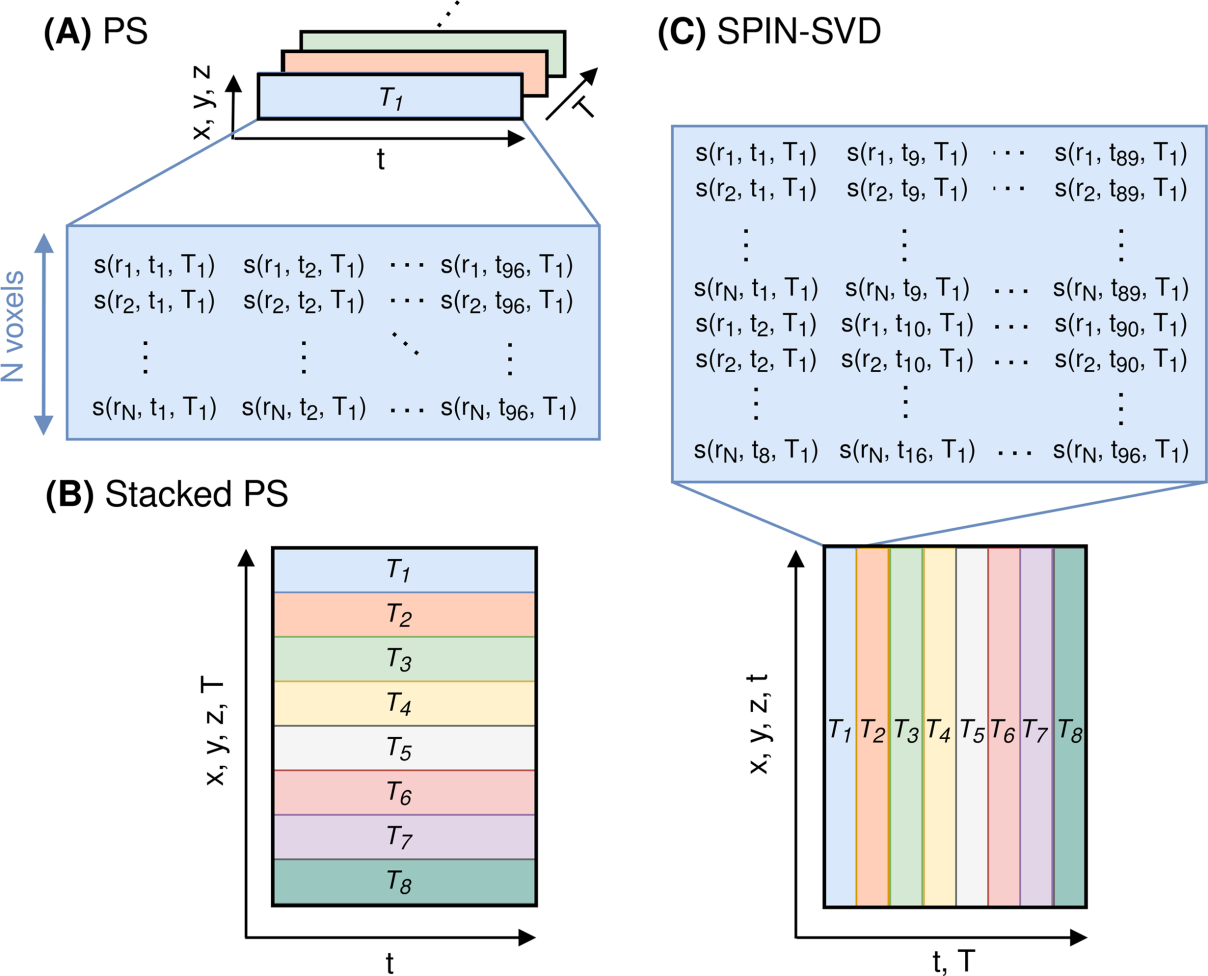
Data reshaping methods: (A) PS: Each ^2^H‐MRSI repetition (*T*_1_, *T*_2_, …, *T*_8_) is reshaped into a Casorati matrix, where each row represents the time‐domain signal from a single spatial location. The matrix can be formed from the entire volume (Global PS) or from patches in voxel space (Local PS). Repetitions are denoised separately. (B) Stacked PS: The Casorati matrices of all repetitions for the full volume are stacked along the spatial dimension, forming a single matrix for low‐rank denoising. (C) SPIN‐SVD: The time and repetition dimensions are not swapped before reshaping, resulting in partial mixing of the time dimension with both the spatial and repetition dimensions. Each column in the resulting matrix contains eight consecutive time points across all spatial locations for a single repetition. This arrangement leverages the strong correlation between the earliest points of the time domain signal—where the signal is highest—across repetitions, because every 12th column of the matrix contains the first eight time points for all spatial locations for a given repetition. The PS and SPIN‐SVD matrix structures are expanded for clarity.

Accordingly, the Casorati matrix of noiseless MRSI data—formed by concatenating the spatial dimensions so that each row represents the spectrum of a given voxel (see Figure 1)—is low rank, with rank L. This allows the noiseless signal to be approximated using a rank‐L approximation of the Casorati matrix derived from the noise‐contaminated measured MRSI data.

PS‐based denoising can be applied either to the entire volume (Global PS) or using a patch‐wise approach (Local PS) [26].

## 3 Methods

In this study, four denoising methods take the ‘matricisation’ approach and two are HOSVD‐based. Here, a brief overview of these methods is provided. More detailed descriptions of the Global PS, GL‐HOSVD and tMPPCA algorithms are given by Nguyen et al. [18], Zhang et al. [19] and Olesen et al. [21], respectively. All denoising methods were implemented in MATLAB R2017b (MathWorks, Natick, MA, USA).

### 3.1 Global PS

While the PS model applies to MRSI data acquired at a single time point, dynamic ^2^H‐MRSI consists of multiple ^2^H‐MRSI repetitions acquired over time to capture changes in the metabolite signal amplitudes following ^2^H‐labelled substrate administration. Thus, Global PS denoising was performed separately on each ^2^H‐MRSI repetition, using an L=6 hard‐thresholded SVD.

The choice of L was guided by the distribution of singular values for in vivo data, as well as the expected number of spectral components [29]. Theoretically, L is constrained by the number of distinct spectral components and tissue types of $\rho \left(r,f\right)$ [18]. Brain ^2^H spectra following administration of [6,6’‐^2^H_2_]‐glucose contain three to four detectable spectral peaks (depending on the presence of a detectable Lac peak), making four the minimum useable L. In practice, however, L is expected to be slightly higher than the number of spectral components, owing to the effects of tissue‐specific *T*_2_ relaxation and *B*_0_ inhomogeneities. Furthermore, use of an L slightly larger than needed is preferable to avoid potential erroneous loss of signal information [29]. This led to the use of L=6 for Global PS denoising; however, different L ranging 6 to 80 were implemented and the preservation of spatial features were analysed as well as visual assessment of the metabolite maps (see Figure S1).

### 3.2 Local PS

Like Global PS, Local PS denoising was performed separately for each dynamic ^2^H‐MRSI repetition. In the Local PS approach, the ^2^H‐MRSI volume is divided into patches in image space, and denoising is performed separately for each patch. The patch size and step size—both user‐specified parameters—determine the spatial extent of each patch and the degree of overlap between neighbouring patches. Voxels that appear in multiple overlapping patches have their multiple denoised signals averaged.

Because each patch can contain the same number of spectral components and tissue types as the full volume, the same SVD threshold as for Global PS (L=6) was applied to each patch. To evaluate the effect of patch size on denoising performance, simulations were performed across a range of patch sizes (see Figure S2), indicating a trade‐off between denoising strength and preservation of spatially localised features. A patch size of 5 × 5 × 5 was used as a compromise between these factors, with a step size of 1.

### 3.3 Stacked PS

For the Stacked PS method, the five‐dimensional (5D) dynamic ^2^H‐MRSI data—comprising three spatial dimensions $\left(x,y,z\right)$, one spectral dimension $\left(t\right)$ and one repetition dimension $\left(T\right)$—were formed into a single matrix by concatenating the Casorati matrices for all ^2^H‐MRSI repetitions along the voxel dimension, as shown in Figure 1. This was achieved by permuting the spectral and temporal dimensions then reshaping the data to have dimensions $\left[x\cdot y\cdot z\cdot T,t\right]$. Hard thresholding with L=8 was used for noise removal, assuming that two additional components are required compared with PS to describe the temporal variation.

### 3.4 SPIN‐SVD

SPIN‐SVD—a novel variation of the PS denoising method—is introduced in this study. Here, the full 5D array is unfolded into a matrix without permuting the spectral and temporal dimensions before reshaping. As shown in Figure 1, this results in a partial mixing of the spectral dimension with both the spatial and temporal dimensions. Each column of the matrix contains $N_{t}$ sequential FID points of all voxels for a given repetition, where $N_{t}$ is an additional parameter of the method. The SPIN‐SVD arrangement leverages the strong correlation between the early FID points, where the signal is highest, as there is one column per repetition containing the first $N_{t}$ FID points of all voxels (see Figure S3E). As shown in Figure 1, $N_{t}=8$ was used, meaning the matrix size for Stacked PS and SPIN‐SVD were the same. Denoising performance was assessed over a range of possible $N_{t}$ (see Figures S3A–D), indicating a trade‐off between residual noise (which increases with $N_{t}$) and preservation of spatial signal variations (which also increase with $N_{t}$). Note that for each repetition to have a column containing the first $N_{t}$ FID points, the total number of FID points must be exactly divisible by $N_{t}$. In practice, this can be achieved by truncating the time‐domain signal to be an exact multiple of $N_{t}$. As for Stacked PS, noise removal was performed using hard thresholding with L=8.

### 3.5 GL‐HOSVD

The denoising algorithm used by Kim et al. [20] for hyperpolarised ^13^C MR imaging was adapted for this study for application to 5D data so that the natural tensor structure of the dynamic ^2^H‐MRSI data could be retained.

The algorithm has two main stages:

1. A prefiltering ‘global’ step: HOSVD is applied to the whole data set prior to hard thresholding of the core tensor, with the threshold scaled by the parameter *k*_global_.
2. A patch‐based ‘local’ step: Patches of the data within a search window are arranged into a tensor based on their similarity to a reference patch in the search window centre. HOSVD is applied to the constructed tensor, and hard thresholding of the core tensor is applied, scaled by parameter *k*_local_.

Kim et al. previously optimised the five GL‐HOSVD parameters (*k*_global_, patch size, search window size, step size, *k*_local_) for hyperpolarised ^13^C MR imaging, and the same parameters have been applied for static ^2^H‐MRSI data [20, 24]. The same parameters were used for this study except for the patch size, which was extended from a 2D patch of 5 × 5 to a 3D patch of 5 × 5 × 5.

### 3.6 tMPPCA

tMPPCA is another patch‐based denoising method using HOSVD [21]. Like GL‐HOSVD, patches are specified in voxel space by the user. However, no global denoising step is applied, and patches are denoised separately rather than being arranged into a modified tensor. Marchenko–Pastur principal component analysis (MPPCA) is applied to each flattening of the tensor patch, which uses noisy matrix theory to objectively estimate the number of signal‐carrying components [30, 31]. Soft thresholding is applied to each flattening using the estimated rank, meaning that the noise contribution successively reduces as the tensor indices are cycled through. A step length determines the extent of overlap of the patches, and for voxels belonging to multiple patches, an average is generated over the multiple estimates.

As for GL‐HOSVD, a patch size of 5 × 5 × 5 was used for denoising. For each patch, a tensor was formed with the spectral data along the first dimension, then the three spatial dimensions, and the temporal data along the last dimension.

### 3.7 In Vivo Acquisition

The study was approved by the local ethics committee of the Medical University of Vienna and followed the guidelines of the Declaration of Helsinki. Dynamic ^2^H‐MRSI brain data were acquired for a healthy volunteer on a 7T MR system (Magnetom dot Plus, Siemens, Germany) using a quadrature dual‐tuned ^2^H/^1^H birdcage head coil (Stark Contrasts MRI Coils Research, Germany). Eight ^2^H‐MRSI scans were acquired between 14 and 63 min after oral administration of 0.8 g/kg body weight of [6,6′‐^2^H_2_]‐glucose. These were acquired using an FID‐based sequence with a 3D density‐weighted concentric ring trajectory readout [15, 32] and a non‐localised rectangular excitation pulse (pulse duration = 500 μs, flip angle = 86°, TR = 290 ms, acquisition delay = 2 ms, FoV = 200 × 200 × 192 mm^3^, matrix = 22 × 22 × 21, number of rings = 43, number of partitions = 43, bandwidth = 380 Hz, number of spectral points = 96, acquisition time per scan = 7 min). The rings and partitions were distributed to approximate a 3D‐Hamming filter. Reconstructed data formed a 22 × 22 × 21 × 96 × 8 tensor $\left(x,y,z,t,T\right)$ with nominal spatial resolution 9.1 × 9.1 × 9.1 mm^3^. Following acquisition of ^2^H‐MRSI data, an anatomical *T*_1_‐weighted image was acquired using an MP2RAGE sequence (FOV = 165 × 220 × 220 mm^3^, matrix size = 144 × 192 × 192, spatial resolution = 1.15 × 1.15 × 1.15 mm^3^, TR = 3930 ms, TI1 = 850 ms, TI2 = 3400 ms, TE = 3.28 ms, acquisition time = 9.29 min).

### 3.8 Simulated Dynamic ^2^H‐MRSI

An anatomical *T*_1_‐weighted image and a *B*_0_ map, measured in one healthy volunteer, served as the basis for the numerical phantom simulation. The *T*_1_‐weighted image was acquired using an MP2RAGE sequence with the following parameters: TR = 5000 ms, TE = 4.13 ms, TI1 = 700 ms, TI2 = 2700 ms, FOV = 168 × 240 × 240 mm^3^, matrix = 224 × 320 × 320, and spatial resolution = 0.75 × 0.75 × 0.75 mm^3^. The parameters of the *B*_0_ acquisition were TR = 13 ms, TE1 = 1.9 ms, TE2 = 3.8 ms, TE3 = 5.7 ms, TE4 = 7.6 ms, TE5 = 9.5 ms, FOV = 225 × 225 × 224 mm^3^, matrix = 112 × 112 × 112, and spatial resolution = 2 × 2 × 2 mm^3^. The ROMEO method with temporal unwrapping and temporal voxel‐wise fitting of the phase was used to construct a *B*_0_ map from the acquired data [33]. The map was converted to Hz based on the ^2^H gyromagnetic ratio.

Dynamic ^2^H‐MRSI brain simulations were generated using an in‐house MATLAB script (R2017b). Probability maps for grey matter (GM), white matter (WM) and cerebrospinal fluid (CSF) were obtained from FAST segmentation of the *T*_1_‐weighted image and formed the spatial basis of the simulated data [34]. Quantum‐simulated HDO (4.8 ppm), Glc (3.9 ppm) and Glx (2.4 ppm) signals were truncated in the spectral and temporal domains to match the bandwidth and spectral resolution, respectively, of the in vivo ^2^H‐MRSI data [35]. J‐couplings were not considered in the simulation, as the broad linewidths observed in deuterium spectra render the effects unresolvable. Tissue‐specific *T*_2_‐relaxation times were applied to the simulated signals to approximate in vivo lineshapes [36, 37]. The simulated tissue‐specific signals were scaled with the corresponding tissue probability maps to mimic the in vivo spatial distributions of metabolite concentrations [15, 38]. Eight ^2^H‐MRSI repetitions were simulated, with HDO and Glx concentrations increasing linearly over time, and Glc following an exponential relaxation curve to approximate in vivo dynamics [15]. The formulas used to compute the tissue‐specific metabolite scaling factors for each repetition are provided in Table S1.

The *B*_0_ map was resampled to match the FOV and resolution of the *T*_1_‐weighted image and applied to the phantom in image space, incorporating frequency shift effects that are present for in vivo acquisitions. To evaluate the performance of the denoising methods under different levels of *B*_0_ inhomogeneity, simulated data were generated with the *B*_0_ map being multiplied by scalings of *λ* = 0, 0.5, 1, 1.5, and 2 before application to the numerical phantom (i.e., *B*_0_^scaled^ = *λ* *·B*_0_).

After application of the scaled *B*_0_ maps, the data were truncated in *k*‐space to match the in vivo spatial resolution (matrix size = 22 × 22 × 21, spatial resolution = 9.1 × 9.1 × 9.1 mm^3^). Frequency correction was applied to the downsampled data before denoising, meaning that *λ* predominantly affects the linewidth due to intravoxel *B*_0_ variations in the downsampled data.

To evaluate the performance of the denoising methods at different noise levels, random complex Gaussian noise was added to the truncated *k*‐space data to simulate data with SNR_HDO_ = 5, 9, 15, 20, 25 and 30, where SNR_*k*_ was defined as the mean absolute height of peak *k* (either HDO, Glc or Glx) divided by the standard deviation of the real noise signal. SNR_HDO_ = 9 corresponds to the estimated in vivo noise level. Following noise addition, a 3D Hamming filter was applied to simulate the point spread function of the hamming‐weighted in vivo data. For all *λ* ≠ 1, SNR_HDO_ = 9 was used.

Noiseless data were processed (see section ‘Data Processing’) for each *λ* to serve as a gold standard for evaluation of the denoising methods.

### 3.9 Simulated Lesion

The ^2^H‐MRSI brain simulation with noise and *B*_0_ inhomogeneity levels reflecting the in vivo data (SNR_HDO_ = 9 and *λ* = 1) was modified to include a lesion with altered metabolism. This enabled evaluation of denoising methods in a scenario with significant focal metabolic alteration, as encountered, for example, in brain tumours [1]. A mask was created at the same resolution as the *T*_1_‐weighted image, featuring a spherical lesion with a 13.5‐mm radius. GM, WM and CSF probabilities within the lesion were set to 0. Simulated HDO, Glc, Glx and Lac signals were generated as before, but with a wider spectral bandwidth of 476 Hz to prevent truncation of the Lac peak, and 120 spectral points to maintain a similar spectral resolution. The Glx signal inside the lesion was set to 10% of the GM signal for all repetitions, while a Lac signal within the lesion was added with 25% higher signal than the GM Glx signal (see Table S1). The phantom was downsampled to match the in vivo resolution (as described in section ‘Simulated Dynamic ^2^H‐MRSI’).

Pathologically driven metabolic alterations—such as steep spatial gradients in the Lac signal around the edges of the tumour—could increase the rank L of the underlying signal compared with data of healthy subjects. However, in a realistic clinical scenario, the presence or spatial extent of such alterations may not be known a priori. In this context, it is preferable to overestimate L to avoid suppressing clinically relevant metabolite signal variations, even at the cost of slightly reduced noise suppression. Therefore, for the lesion simulation, L was increased to 12 for Local and Global PS and 14 for Stacked PS and SPIN‐SVD.

### 3.10 Data Processing

Reconstruction of the in vivo dynamic ^2^H‐MRSI was carried out using a custom post‐processing pipeline (MATLAB R2017), including a spectral off‐resonance correction along the circle trajectory [39], discrete Fourier transform along the *x*‐*y* directions, and discrete Fourier transform along the *z* direction. Voxel‐wise alignment of the HDO peak was performed. As a result, *B*_0_ inhomogeneity effects were confined to intravoxel variations, which cannot be corrected in post‐processing. Spectral fitting of in vivo and synthetic data was carried out voxel‐wise using LCModel v6.3, with a simulated basis set that included ^2^H resonances of Lac (1.3 ppm), Glx (2.4 ppm), Glc (3.9 ppm) and HDO (4.8 ppm), and did not account for J‐coupling. All basis metabolite signals had a 10‐Hz linewidth, except Lac, which was simulated with a 5.2‐Hz linewidth due to the longer *T*_2_ relaxation constant [1]. Denoising methods for both in vivo and simulated data were applied immediately prior to spectral fitting.

### 3.11 Evaluation

The generation of realistic ^2^H‐MRSI simulations enabled a direct comparison between denoised data and a noiseless gold standard. Thirty noise realisations were added to the noiseless simulation and denoised using each of the six methods prior to spectral fitting. Denoising performance was evaluated through visual assessment of the fitted HDO, Glc and Glx maps compared with the corresponding gold standard maps. Maps of the mean and variance of the difference to the gold standard maps were also computed.

For each simulated noise and *B*_0_ inhomogeneity level, a spectral RMSE against the noiseless simulation was calculated for each dynamic repetition of a single noise instance [26]:

(6) $$\mathrm{RMS}E_{\text{spectral}}=\sqrt{\sum_{r}^{N}\sum_{t}^{M}\frac{{\left(s\left(r,t\right)-\hat{s}\left(r,t\right)\right)}^{2}}{s{\left(r,t\right)}^{2}}},$$

where N is the number of voxels, M is the number of FID points and s and $\hat{s}$ are the noiseless and denoised time‐domain signals, respectively, at spatial location r and time t.

Following spectral fitting, concentration RMSEs were calculated for each repetition using the gold standard metabolite maps generated from the noiseless simulations [26]:

(7) $$\mathrm{RMS}E_{\text{concentration}}=\sqrt{\sum_{k=1}^{3}\sum_{r}^{N}\frac{{\left(c_{k}\left(r\right)-{\hat{c}}_{k}\left(r\right)\right)}^{2}}{c_{k}{\left(r\right)}^{2}}},$$

where $c_{k}$ and ${\hat{c}}_{k}$ are the amplitudes of the kth metabolite map (HDO, Glc, Glx) at location r, fitted from the noiseless and denoised data, respectively.

For the lesion simulations, Lac signal preservation was quantified by computing a weighted average of the lesion's Lac signal. The high‐resolution lesion map was downsampled to match the ^2^H‐MRSI spatial resolution, and the resulting map was thresholded between 0.1 and 1. The weighted mean Lac signal was calculated over included voxels, where the weightings were given by the voxels' corresponding lesion probability map value.

Preservation of the GM‐to‐WM Glx contrast, which has been observed in vivo [15], was evaluated for both simulated and in vivo data. For the final ^2^H‐MRSI repetition, the Glx signals were averaged over GM‐ and WM‐dominated voxels using thresholds of 0.5 and 0.6 for the GM and WM tissue probability maps, respectively. The ratio of the mean GM‐to‐WM Glx signals was then calculated, yielding an ‘apparent’ GM‐to‐WM Glx contrast.

‘Pure’ GM and WM metabolite concentrations ($C_{k,\mathrm{GM}}$ and $C_{k,\mathrm{WM}}$) were calculated to reduce the partial volume effects from the finite voxel size. The metabolite concentration at each voxel was modelled as a linear combination of GM, WM and CSF contributions:

(8) $${\hat{c}}_{\mathrm{Glc}}\left(r\right)=f_{\mathrm{GM}}\left(r\right)C_{\mathrm{Glc},\mathrm{GM}}+f_{\mathrm{WM}}\left(r\right)C_{\mathrm{Glc},\mathrm{WM}}+f_{\mathrm{CSF}}\left(r\right)C_{\mathrm{Glc},\mathrm{CSF}}+\varepsilon ,$$

(9) $${\hat{c}}_{\mathrm{Glx}}\left(r\right)=f_{\mathrm{GM}}\left(r\right)C_{\mathrm{Glx},\mathrm{GM}}+f_{\mathrm{WM}}\left(r\right)C_{\mathrm{Glx},\mathrm{WM}}+f_{\mathrm{CSF}}\left(r\right)C_{\mathrm{Glx},\mathrm{CSF}}+\varepsilon ,$$

where ${\hat{c}}_{\mathrm{Glc}}\left(r\right)$ and ${\hat{c}}_{\mathrm{Glx}}\left(r\right)$ are the Glc and Glx signals for a voxel at spatial location r; $f_{\mathrm{GM}}\left(r\right)$, $f_{\mathrm{WM}}\left(r\right)$ and $f_{\mathrm{CSF}}$(r) are the voxels' GM, WM and CSF fractions, respectively; and ε is the residual error. Least‐squares fitting of the tissue probability maps was applied to the in vivo metabolite maps to estimate $C_{\mathrm{Glc},\mathrm{GM}}$, $C_{\mathrm{Glc},\mathrm{WM}}$, $C_{\mathrm{Glx},\mathrm{GM}}$ and $C_{\mathrm{Glx},\mathrm{WM}}$ for each repetition.

With no in vivo gold standard, denoising performance for in vivo dynamic ^2^H‐MRSI data was evaluated through visual assessment of the HDO, Glc and Glx maps. $C_{\mathrm{Glc},\mathrm{GM}}$, $C_{\mathrm{Glc},\mathrm{WM}}$, $C_{\mathrm{Glx},\mathrm{GM}}$ and $C_{\mathrm{Glx},\mathrm{WM}}$ were plotted against time to assess the impact of the denoising methods on metabolite kinetics.

For all four matricisation methods (Global PS, Local PS, Stacked PS, SPIN‐SVD), the user‐defined rank has important implications for the denoising performance. An overestimated rank would reduce the denoising effectiveness (i.e., higher residual noise would remain), while an underestimated rank could cause spatial blurring or line broadening [18]. To investigate the impact of rank selection on the denoising performance, Global PS, Local PS, Stacked PS and SPIN‐SVD were performed on both simulated and in vivo data using a range of ranks $L\in \left[6,96\right]$. For each method, the pure Glx GM‐to‐WM ratio was calculated, and the fitted HDO, Glc and Glx maps were assessed.

## 4 Results

### 4.1 Simulated Dynamic ^2^H‐MRSI Data

**FIGURE 2:**
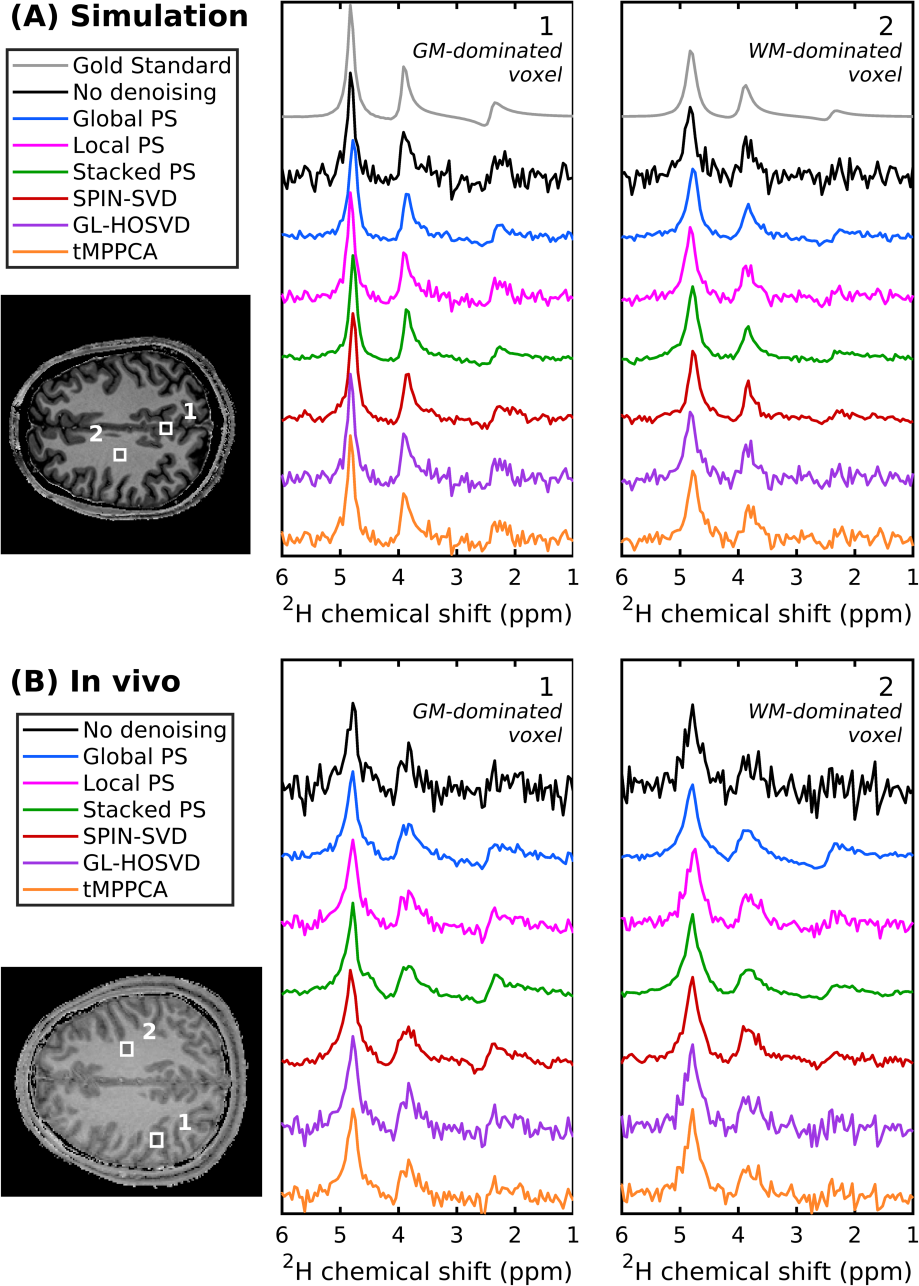
Sample spectra for (A) simulated and (B) in vivo dynamic ^2^H‐MRSI data after applying different denoising methods. Spectra for the final ^2^H‐MRSI repetition are shown for two voxels containing predominantly GM (1) and WM (2). For simulated data, the noiseless spectra are also shown. Voxel locations are indicated on anatomical *T*_1_‐weighted images, which were (A) used for simulation and (B) acquired immediately after the in vivo dynamic ^2^H‐MRSI scan. In both simulated and in vivo data, spectra denoised using GL‐HOSVD and tMPPCA exhibit higher residual noise compared with Global PS, Stacked PS and SPIN‐SVD, with Local PS showing intermediate residual noise between these two groups.

Figure 2A shows representative spectra from GM‐ and WM‐dominated voxels for the noiseless ^2^H‐MRSI simulation, as well as for the simulations where different denoising methods were applied. All spectra have a *B*_0_‐inhomogeneity scaling of *λ* = 1, and the denoised spectra had an input SNR_HDO_ = 9. For both voxels, Global PS, Stacked PS and SPIN‐SVD decreased the noise variance the most. While Local PS resulted in higher residual noise than the other matricisation methods, the spectra following GL‐HOSVD and tMPPCA have the highest residual noise. Spectral artefacts were observed in several voxels following SPIN‐SVD denoising (Figure S4). The impact of denoising on lineshape was assessed by calculating the mean LCModel‐reported FWHM (across all fitted voxels) for the final ^2^H‐MRSI repetition. All denoising methods yielded a slightly reduced linewidth compared with non‐denoised data, which had a mean (± standard deviation) FWHM of 0.13 ± 0.10 ppm. The mean FWHM were 0.10 ± 0.11, 0.11 ± 0.09, 0.09 ± 0.10, 0.10 ± 0.11, 0.12 ± 0.10 and 0.12 ± 0.10 ppm for data denoised with Global PS, Local PS, Stacked PS, SPIN‐SVD, GL‐HOSVD and tMPPCA, respectively.

**FIGURE 3:**
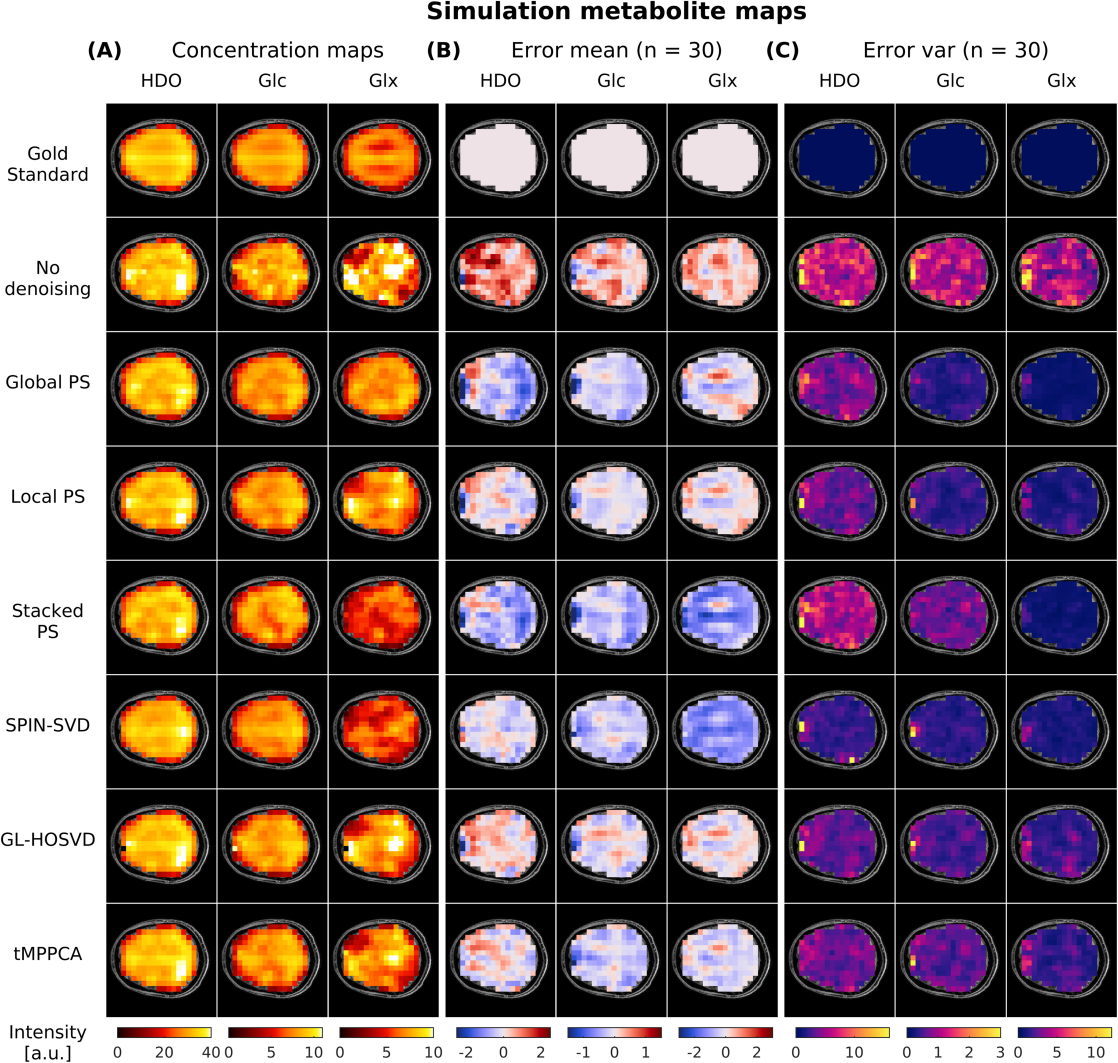
(A) Fitted maps of HDO, Glc and Glx after applying different denoising methods to the ^2^H‐MRSI simulation for a single noise instance. Voxel‐wise (B) mean and (C) variance of the error compared with the noiseless gold standard maps, calculated over 30 noise realisations. All maps show the final repetition of the simulated ^2^H‐MRSI data. The input Gaussian noise amplitude (before denoising) corresponds to spectral SNR_HDO_ = 9, SNR_glc_ = 4 and SNR_glx_ = 1.7. SPIN‐SVD performed best for HDO, resulting in low bias and variation, but introduced bias for Glx as shown by the overall negative mean error map. SPIN‐SVD and Local PS resulted in low bias and variance for Glc. All methods introduced distinct residual patterns in the Glx mean error maps, indicating GM‐to‐WM Glx contrast loss. GL‐HOSVD and tMPPCA resulted in low bias but higher error variance for Glx.

Figure 3A compares the HDO, Glc and Glx maps fitted from noiseless, noise‐added and denoised ^2^H‐MRSI simulations (*λ* = 1 and input SNR_HDO_ = 9 before denoising) for a single noise realisation. Maps of the mean and variance of the error compared with the gold standard maps over 30 noise realisations are displayed in Figures 3B,C, respectively. SPIN‐SVD performed best for HDO, showing a closer resemblance to the gold standard map and lower values of the mean and variance maps compared with other methods. SPIN‐SVD and Local PS performed well for Glc, resulting in low mean and variance of error. Local PS resulted in lower mean errors than Global PS for all three metabolites, with only slightly higher error variances for Glx. For Glx, the lowest SNR metabolite of the simulation, all methods introduced distinct patterns in the mean error maps. For Global PS, Local PS, GL‐HOSVD and tMPPCA, the mean error values were negative in GM‐dominated regions (where simulated Glx concentration was higher) and positive in WM‐dominated regions (where the simulated Glx concentration was lower), indicating that the simulated GM‐to‐WM Glx contrast was reduced following denoising. Stacked PS led to an overall supressed Glx signal, indicated by the negative mean error throughout most of the displayed slice. For SPIN‐SVD, the Glx signal was also suppressed, as illustrated by the overall negative mean error, and suppression was more severe in the GM than WM. GL‐HOSVD and tMPPCA produced similar maps and mostly preserved the spatial distributions of HDO and Glc. The distributions of voxel‐wise metabolite errors (Figure S5) for all six denoising methods showed a reduced error spread for all metabolites. For Glx, the error distribution was narrowest following Global PS and SPIN‐SVD, while SPIN‐SVD also produced a narrow error distribution for HDO and Glc in line with the closer resemblance to the gold standard maps.

The apparent GM‐to‐WM Glx signal ratios were calculated by averaging the Glx signals of the final repetition across voxels within the GM and WM masks. The resulting ratios were (mean ± standard deviation over noise instances) 1.39 for gold standard data, 1.27 ± 0.10 for non‐denoised data and 0.99 ± 0.05, 1.14 ± 0.10, 0.90 ± 0.05, 1.39 ± 0.08, 1.16 ± 0.11 and 1.23 ± 0.08 for data denoised with Global PS, Local PS, Stacked PS, SPIN‐SVD, GL‐HOSVD and tMPPCA, respectively. To account for the impact of partial volume effects, ‘pure’ GM and WM metabolite signals were determined by applying least‐squares fitting of the tissue probability maps to the metabolite maps. The pure GM‐to‐WM Glx ratios for the final repetition were 4.97 for gold standard data, 3.66 ± 1.22 for the non‐denoised data and 1.67 ± 0.16, 2.67 ± 0.67, 1.59 ± 0.17, 4.36 ± 0.66, 2.96 ± 0.78 and 3.39 ± 0.75 for data denoised with Global PS, Local PS, Stacked PS, SPIN‐SVD, GL‐HOSVD and tMPPCA, respectively.

To investigate if rank underestimation was the cause of GM‐to‐WM Glx contrast loss, the pure GM‐to‐WM Glx signal ratio was plotted for a range of different selected ranks for Global PS, Local PS, Stacked PS and SPIN‐SVD, as shown in Figure S3. For Global PS and Stacked PS, the loss of GM‐to‐WM Glx contrast persisted over a range of selected ranks, but became more severe as a lower rank was selected. For tMPPCA, the ranks for different flattenings are objectively estimated using noisy matrix theory. The voxel‐wise mean estimated rank for each tensor flattening is shown in Table S2. Across different simulated SNR and *λ* values, the mean estimated rank for the t‐mode flattening ranged from 43.93 ± 0.58 to 45.60 ± 0.87, exhibiting a slight upward trend with increasing SNR and *λ*. Notably, for the T‐mode flattening, the mean estimated rank was 8.00 ± 0.00 across all SNR and *λ*. This indicates that the algorithm did not identify any noise components to be removed along the T dimension, because only eight repetitions were simulated.

**FIGURE 4:**
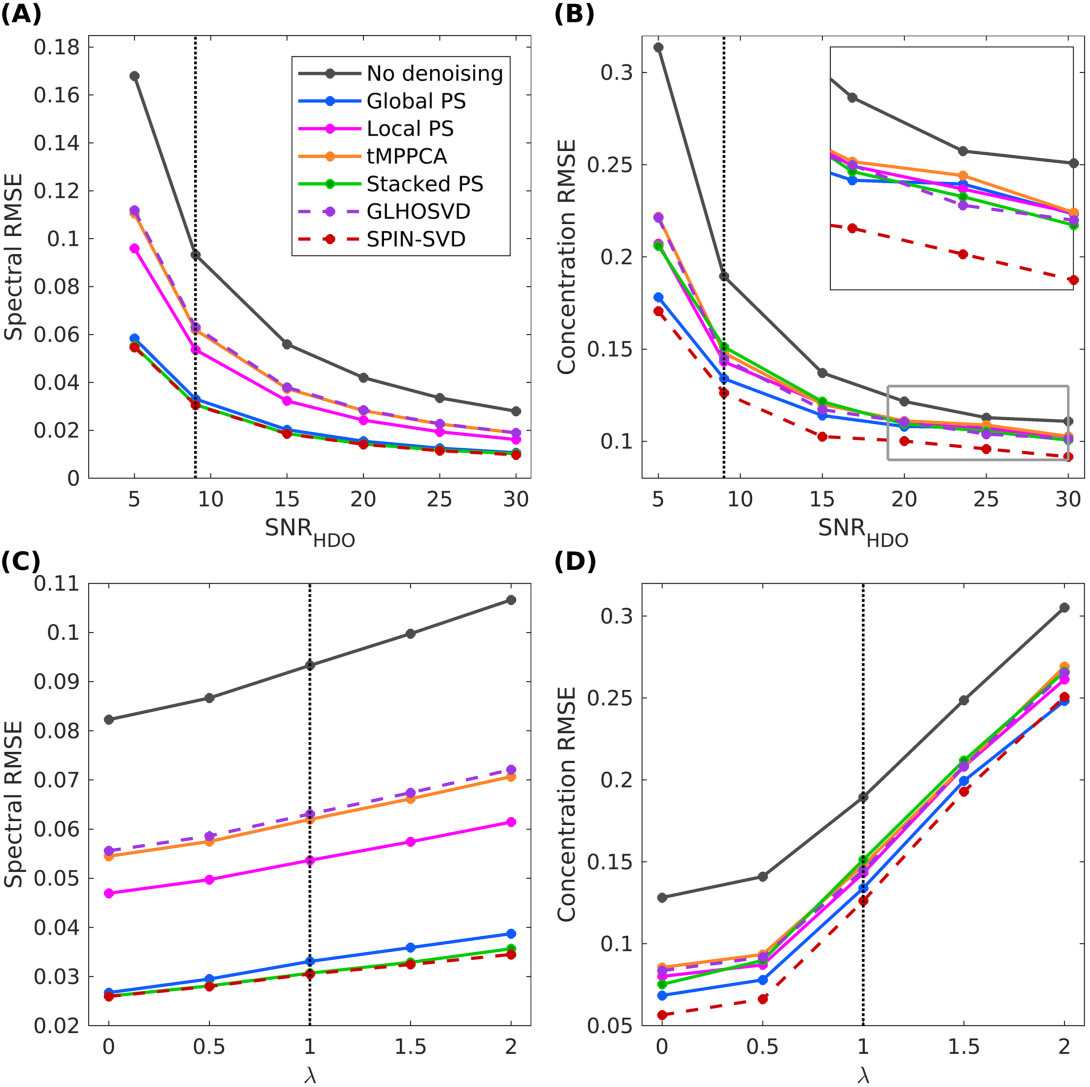
(A) Spectral RMSE and (B) concentration RMSE for different denoising methods, plotted as a function of simulated SNR_HDO_. (C) Spectral RMSE and (D) concentration RMSE as a function of *λ*. All simulations for different SNR_HDO_s were performed at the in vivo *B*_0_ level (*λ* = 1), and simulations to investigate the effect of *B*_0_ inhomogeneities were performed at the estimated in vivo noise level (SNR_HDO_ = 9). The black dotted lines indicate the input SNR_HDO_ and *λ* used for the simulated maps of Figure 3. Although PS, Stacked PS and SPIN‐SVD resulted in similar spectral RMSEs, SPIN‐SVD resulted in a consistently lower concentration RMSE across all investigated SNRs and all but the highest *λ*. GL‐HOSVD and tMPPCA led to similar spectral RMSEs, which were slightly higher than Local PS and much higher than the other denoising methods. However, the concentration RMSEs following Local PS, GL‐HOSVD and tMPPCA were comparable to Stacked PS.

For each simulated SNR_HDO_ and *λ*, the denoising methods were applied, and the spectral and concentration RMSEs were computed for a single noise instance. These RMSEs were then averaged over all repetitions, yielding a single spectral and concentration RMSE per denoising method per simulation. Figure 4A,B show the spectral and concentration RMSEs, respectively, plotted against simulated SNR_HDO_, while Figure 4C,D show the same metrics plotted against simulated *λ*. All denoising methods reduced both the spectral and concentration RMSE across the full range of simulated SNR_HDO_ and *λ* values. For the simulation with SNR_HDO_ and *λ* corresponding to the in vivo level, the concentration RMSEs were 29.3%, 24.4%, 20.2%, 33.4%, 23.5% and 21.9% lower following denoising with Global PS, Local PS, Stacked PS, SPIN‐SVD, GL‐HOSVD and tMPPCA, respectively. For all simulated *λ* and SNR_HDO_ values, Global PS, Stacked PS and SPIN‐SVD produced much lower spectral RMSEs than GL‐HOSVD and tMPPCA, and slightly lower than Local PS, in agreement with the perceived spectral denoising shown in Figure 2A. Interestingly, this did not translate to much lower concentration RMSEs following LCModel fitting. GL‐HOSVD, tMPPCA and Local PS led to concentration RMSEs comparable to those of Global PS and Stacked PS. Additionally, despite the fact that the spectral RMSEs for Stacked PS and SPIN‐SVD were comparable, SPIN‐SVD achieved the lowest concentration RMSE across all SNR_HDO_s and at all but the highest *λ*, where Global PS denoising resulted in the lowest concentration RMSE.

### 4.2 Simulated Dynamic ^2^H‐MRSI Lesion Model

**FIGURE 5:**
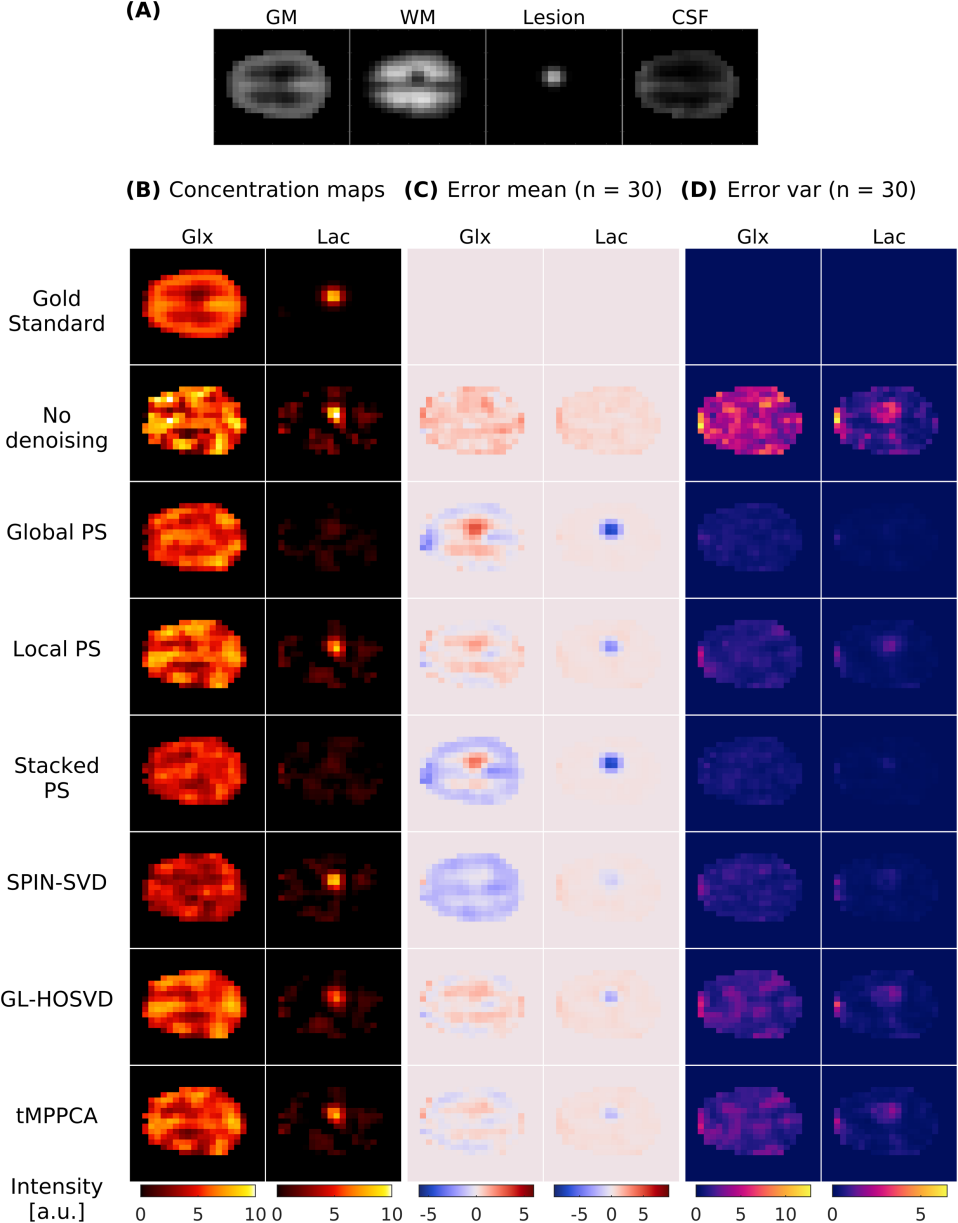
Denoising of a simulated lesion model. (A) GM, WM, Lesion and CSF maps downsampled to match the simulated ^2^H‐MRSI spatial resolution. (B) Maps of Glx and Lac fitted to the ^2^H‐MRSI lesion model with no added noise (gold standard) or with added noise for a single noise instance followed by different denoising methods. Voxel‐wise (C) mean and (D) variance of the error compared with the gold standard maps, calculated over 30 noise instances. All maps correspond to the final repetition. All denoising methods were performed on data with added Gaussian noise corresponding to spectral SNR _HDO_ = 6.4, SNR_Glx_ = 1.5 and SNR_Lac_ = 3.1, where the Lac peak height for the SNR calculation was averaged over voxels with lesion probability > 0.3. SPIN‐SVD best preserved the Lac signal of the lesion but reduced the Glx signal. tMPPCA preserved the Lac signal slightly more than GL‐HOSVD. Following Global PS and Stacked PS denoising, the lesion Lac signal is almost imperceptible. Local PS reduced the lesion Lac signal slightly more than GL‐HOSVD but less than Global and Stacked PS.

Figure 5 shows Glx and Lac maps for the gold standard lesion simulation, the noise‐added lesion simulation (SNR_HDO_ = 9) without denoising and the noise‐added lesion simulations after denoising with the six methods. The gold standard Glx map features a lower signal in the region corresponding to the lesion. Similar to the non‐lesion simulated Glx maps, all methods led to distinct residual patterns in the mean error maps. The Glx mean error maps for Global PS and Stacked PS, have a hotspot coinciding with the lesion, indicating that they did not preserve the lesion's reduced Glx signal. The Local PS, GL‐HOSVD and tMPPCA Glx and corresponding mean error maps are comparable, demonstrating a less pronounced loss of the reduced lesion Glx signal. The residual Glx map following SPIN‐SVD also lacked this pronounced error and displayed a fairly uniform reduction in the Glx signal throughout.

Although the ground truth data contained no Lac signal outside the lesion, the addition of noise resulted in a low, varying level of spurious Lac signal throughout the brain. All five denoising methods effectively suppressed the Lac signal outside the lesion, and no spillover of the lesion's Lac signal into adjacent voxels was observed; however, the Lac signal within the lesion was erroneously reduced to varying extents. Global PS and Stacked PS denoising nearly removed the Lac signal in the lesion entirely, while SPIN‐SVD's Lac map most closely resembled the gold standard. The HOSVD‐based methods also suppressed the lesion Lac signal—GL‐HOSVD more so than tMPPCA—but both retained the signal more than Global PS and Stacked PS. Local PS preserved the lesion Lac signal to a greater extent than Global and Stacked PS, though not as effectively as SPIN‐SVD, GL‐HOSVD and tMPPCA. The weighted Lac signal in the lesion (mean ± standard deviation over noise instances) was 4.23 for the gold standard simulation, 4.74 ± 0.88 for the non‐denoised simulation and 0.68 ± 0.27, 2.66 ± 0.69, 0.59 ± 0.37, 3.68 ± 0.36, 3.52 ± 0.71 and 3.67 ± 0.70 for the simulations denoised with Global PS, Local PS, Stacked PS, SPIN‐SVD, GL‐HOSVD and tMPPCA respectively. SPIN‐SVD most closely approximated the gold standard value, followed by tMPPCA, while Global PS and Stacked PS exhibited the highest deviations from the gold standard value.

### 4.3 In Vivo Dynamic ^2^H‐MRSI Data

Figure 2B shows sample spectra in GM‐ and WM‐dominated voxels following the different denoising methods. In line with the simulated data, Global PS, Stacked PS and SPIN‐SVD decreased the noise variance more than GL‐HOSVD and tMPPCA, with Local PS showing intermediate noise suppression. The voxel‐wise mean FWHM (± standard deviation) for the final ^2^H‐MRSI repetition was 0.22 ± 0.12 ppm for non‐denoised data and 0.22 ± 0.14, 0.21 ± 0.12, 0.19 ± 0.15, 0.21 ± 0.14, 0.21 ± 0.12 and 0.20 ± 0.12 ppm for data denoised with the Global PS, Local PS, Stacked PS, SPIN‐SVD, GL‐HOSVD and tMPPCA approaches, respectively.

**FIGURE 6:**
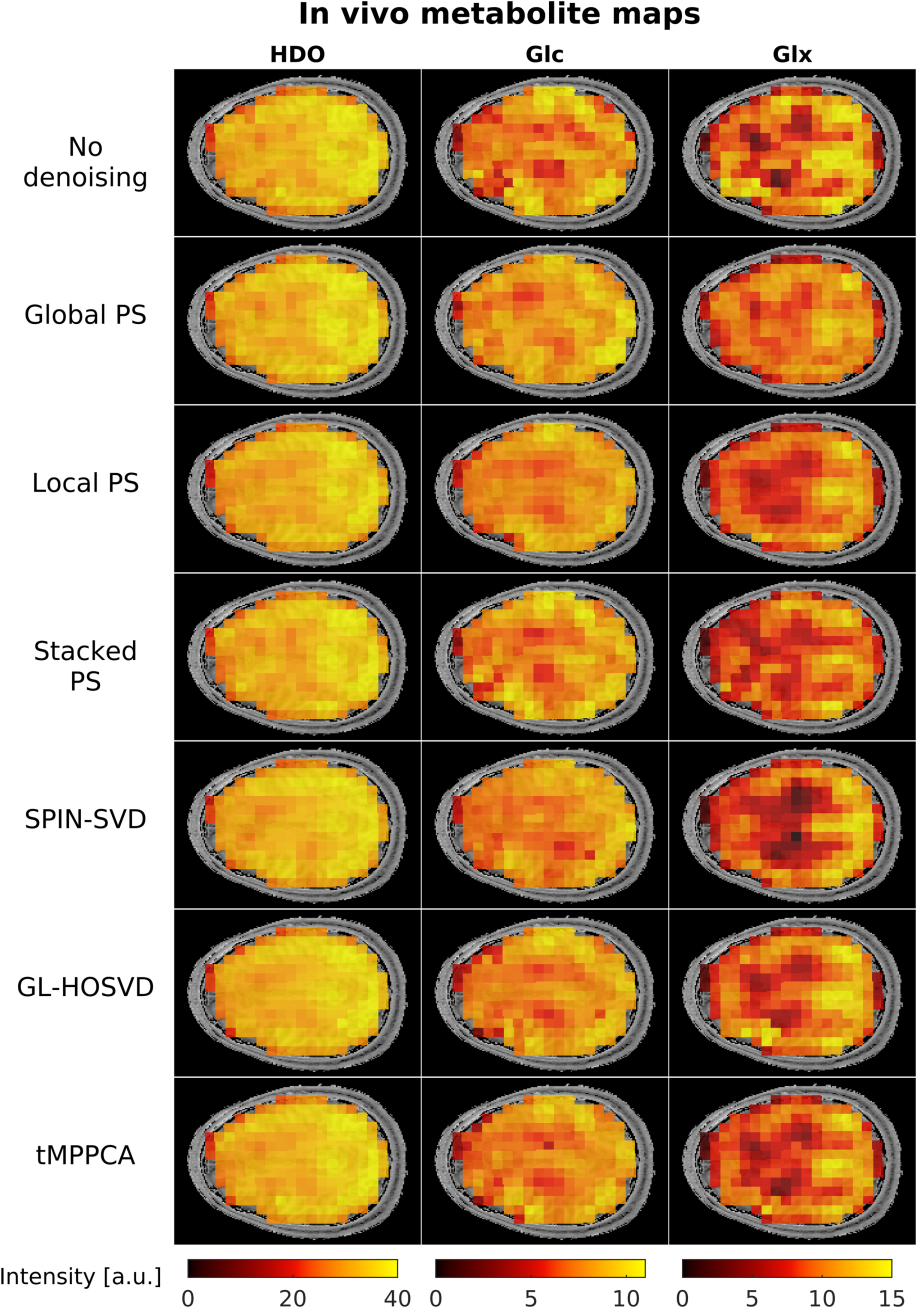
In vivo maps of HDO, Glc and Glx fitted after applying different denoising methods to the dynamic ^2^H‐MRSI data. All maps correspond to the final ^2^H‐MRSI repetition. Differences in denoising performance are most pronounced in the Glx maps, where SPIN‐SVD led to the highest GM‐to‐WM contrast, while Global PS resulted in markedly reduced contrast. Local PS slightly reduced the GM‐to‐WM Glx contrast although not as severely as Global PS. The GL‐HOSVD and tMPPCA maps are similar, both displaying GM‐to‐WM contrast in the Glx map.

Figure 6 compares the in vivo HDO, Glc and Glx maps fitted without denoising and after applying each of the six denoising methods. The HDO maps were similar across denoising methods, although SPIN‐SVD, GLHOSVD and tMPPCA reduced noise variance more noticeably. Differences were more striking between the Glx maps. Consistent with the findings for simulated data, Global PS and Stacked PS denoising reduced the GM‐to‐WM Glx contrast compared with other methods. SPIN‐SVD, GL‐HOSVD and tMPPCA preserved the Glx contrast, while Local PS slightly reduced it. Apparent GM‐to‐WM Glx ratios were calculated using the same thresholds as for the simulated ^2^H‐MRSI data, yielding values of 1.31 ± 0.63 for non‐denoised data and 1.08 ± 0.31, 1.18 ± 0.40, 1.16 ± 0.42, 1.38 ± 0.59, 1.26 ± 0.50 and 1.25 ± 0.52, for data denoised using Global PS, Local PS, Stacked PS, SPIN‐SVD, GL‐HOSVD and tMPPCA, respectively.

**FIGURE 7:**
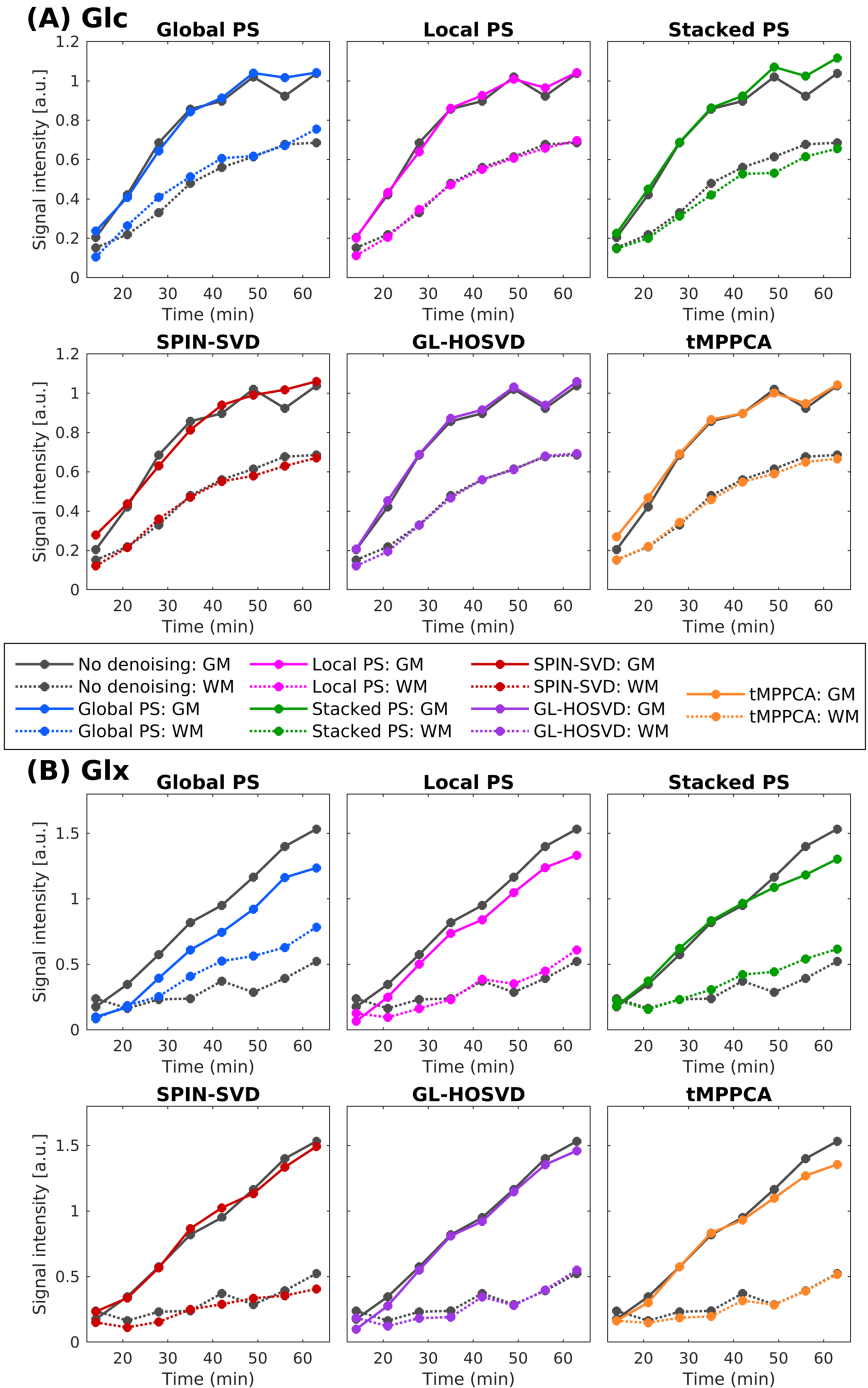
In vivo time‐series of ‘pure’ GM and WM Glc (A) and Glx (B) signals, derived from least‐squares fitting of the in vivo Glc and Glx concentration maps to the tissue probability maps. Stacked PS slightly increased the difference between the pure GM and WM Glc signals while reducing the difference for Glx. Global PS substantially decreased the GM‐to‐WM difference in Glx signals, while Local PS led to a less marked reduction. SPIN‐SVD led to smoothing of both the Glc and Glx time courses. The GL‐HOSVD time courses most closely resembled those of the non‐denoised data, while tMPPCA also closely followed the non‐denoised time courses, except for the pure GM signal in the final three repetitions.

Figure 7 shows the time course of the pure Glc and Glx signals for GM and WM following application of the different denoising methods. Stacked PS slightly increased the difference between pure GM and WM Glc signals, while SPIN‐SVD smoothed the time courses, resulting in a slightly higher pure GM Glc signal for the first repetition compared with the non‐denoised data. The Glc time courses following GL‐HOSVD and tMPPCA closely followed the non‐denoised Glc time course. As with simulated data, no noise components were removed for the T‐mode flattening of the tMPPCA patches (see Table S2). For Glx, both Global PS and Stacked PS decreased the GM‐to‐WM difference, with Global PS showing the most pronounced reduction. Local PS led to slightly lower pure GM Glx signal for all repetitions. GL‐HOSVD and tMPPCA mostly retained the non‐denoised time courses, though tMPPCA slightly reduced the pure GM Glx signal in the final three repetitions. The pure GM‐to‐WM Glx ratio for the final ^2^H‐MRSI repetition was 2.93 ± 0.93 for non‐denoised data and 1.58 ± 0.32, 2.18 ± 0.52, 2.11 ± 0.51, 3.67 ± 1.27, 2.65 ± 0.72 and 2.62 ± 0.75 for data denoised using Global PS, Local PS, Stacked PS, SPIN‐SVD, GL‐HOSVD and tMPPCA, respectively.

## 5 Discussion

In this work, we compared six low‐rank denoising methods for dynamic ^2^H‐MRSI using simulated and in vivo brain data. Overall, SPIN‐SVD outperformed other approaches for denoising dynamic ^2^H‐MRSI simulations, including a lesion model featuring altered metabolism. SPIN‐SVD, GL‐HOSVD and tMPPCA all performed well for denoising in vivo data. Our results indicate that Global PS and Stacked PS are not useful denoising approaches for dynamic ^2^H‐MRSI, as they led to degraded Glx contrast in simulated and in vivo data and the removal of Lac signal in a simulated lesion. Local PS performed better than Global PS both for simulated and in vivo data but did not perform as well as the HOSVD patch‐based methods.

SPIN‐SVD best preserved simulated spatial variations in the HDO, Glc and Glx signals while reducing noise variance, and yielded the lowest concentration RMSE across all simulated noise levels and all but the highest simulated B_0_ inhomogeneity level. Furthermore, SPIN‐SVD best preserved the simulated GM‐to‐WM Glx contrast and focal Lac signal. The $N_{t}$ value was optimised for the data of this study, but we expect the optimal $N_{t}$ to increase with the FID sampling rate. It should be noted that SPIN‐SVD suppressed the Glx signal throughout the simulated volume. Substantially less suppression was observed when a higher Glx SNR was simulated (not shown). However, this indicates that SPIN‐SVD may be less suitable when the measurement goal is absolute quantification of very low SNR metabolites.

GL‐HOSVD and tMPPCA performed similarly, with slightly superior retention of the lesion Lac signal following tMPPCA and slightly better preservation of the in vivo Glx timecourse following GL‐HOSVD. Both methods were expected to introduce bias in very low‐SNR scenarios, because the MP rank selection tends to 1 as signal components become indistinguishable from noise, and the GL‐HOSVD thresholds scale with the noise standard deviation. Accordingly, the concentration RMSEs for GL‐HOSVD and tMPPCA are higher than all other methods for the lowest SNR simulation of the study.

*B*_0_ inhomogeneities are expected to affect the signal rank [40]. Accordingly, tMPPCA was expected to be more versatile than GL‐HOSVD across different *B*_0_ inhomogeneity levels due to its objective rank estimation. However, both methods performed similarly across the range of simulated *B*_0_ inhomogeneity levels, producing similar spectral and concentration RMSEs. Notably, the number of retained components for tMPPCA exhibited minimal variation across the range of B_0_ inhomogeneity levels (see Table S2). This suggests that over the range of *B*_0_ inhomogeneity levels investigated in this study, the rank of ^2^H‐MRSI data remains relatively stable. However, it is important to note that frequency alignment was applied to the spectra as part of the processing pipeline, so only the intra‐voxel line broadening was simulated in this study. If frequency alignment were omitted, the rank may have varied more [30]. Between the two HOSVD‐based methods, we recommend tMPPCA as the superior choice for its objective rank estimation and greater computational efficiency despite similar performances. Dynamic ^2^H‐MRSI is a very new methodology with potential for significant improvements in spatial and temporal resolution, and so computational efficiency will become an increasingly important feature in the future.

Global PS and Stacked PS led to significant degradation of the Glx spatial distribution in both simulated and in vivo data, as well as the disappearance of the focal Lac signal in the lesion simulation. For all matricisation‐based denoising methods, underestimation of rank—which is user‐specified—can lead to erroneous signal loss [18]. However, the loss of GM‐to‐WM Glx contrast persisted over a wide range of selected ranks (10–80) for Global PS and Stacked PS denoising. While Local PS performed significantly better than Global PS, it led to a greater reduction in GM‐to‐WM Glx contrast compared with GL‐HOSVD and tMPPCA for both simulated and in vivo data, as well as to a greater reduction in the lesion Lac signal of lesion simulations.

Of the six approaches investigated, we recommend SPIN‐SVD or tMPPCA for denoising dynamic ^2^H‐MRSI data. SPIN‐SVD outperformed all other methods in ^2^H‐MRSI simulation and performed well for in vivo data. Furthermore, its simple implementation and superior preservation of metabolic alterations make it the favourable method for clinical applications. However, tMPPCA may be more suitable for applications requiring absolute quantification of very low SNR metabolites.

The use of simulated MRSI data is a well‐established method for evaluating the performance of low‐rank denoising methods [15, 24, 26, 31, 41]. However, to the best of our knowledge, this study is the first to validate low‐rank denoising methods using realistic dynamic ^2^H‐MRSI simulations.

Low‐rank denoising methods have been applied to ^2^H‐MRSI data in four studies [6, 15, 24, 41], including one static ^2^H‐MRSI simulation [42]. Kreis et al. used a Tucker decomposition for 5D rank reduction of in vivo dynamic ^2^H‐MRSI of a murine lymphoma model, reporting improved SNR of metabolite maps [6]. The method was also applied to a phantom containing compartments with known concentrations of ^2^H‐labelled metabolites and appears to slightly smear the measured signals outside the compartment boundaries. In our study, two of the methods employed, GL‐HOSVD and tMPPCA, are HOSVD‐based, which is a specific orthogonal form of Tucker decomposition. Neither method caused smearing when applied to a simulated lesion model featuring a focal metabolic alteration. Both GL‐HOSVD and tMPPCA employ patch‐based denoising, supporting the idea that patch‐based methods are superior for preserving local metabolite variations while reducing noise variance.

Christensen et al. compared GL‐HOSVD and tMPPCA across a range of X‐nuclei data. They found similar performances of the two algorithms for in vivo brain ^2^H‐MRSI, with GL‐HOSVD better removing noise around the brain and tMPPCA better revealing signal at the brain periphery [24]. Our study extended this comparison to dynamic ^2^H‐MRSI of the brain using simulated and in vivo data. We also found comparable performances of the two methods, reinforcing their similarity for denoising of ^2^H‐MRSI data, including for dynamic ^2^H‐MRSI, which feature an additional dynamic dimension.

Nam et al. demonstrated the feasibility of low‐rank and subspace model‐based reconstruction for simulated and in vivo ^2^H‐MRSI data of the human liver that were retrospectively undersampled in *k*‐space [41]. In this work, we have further demonstrated that low‐rank‐based reconstruction is feasible for ^2^H‐MRSI accelerated using spatial‐spectral encoding via concentric ring trajectories, as used for the in vivo acquisition.

As well as low‐rank methods, deep‐learning approaches have been applied to enhance the SNR of ^2^H‐MRSI [43, 44]. Li et al. integrated physics‐based subspace modeling and deep learning to improve the sensitivity of dynamic ^2^H‐MRSI [43], enabling the acquisition of rat brain tumour images at 16.4T with a nominal spatial resolution of 1.65 × 1.65 × 4.8 mm^3^ and a temporal resolution of 0.9–1.8 min. Whereas their approach integrates physics‐based prior knowledge, the five denoising methods applied in our study show that high spatiotemporal resolution data can also be attained using a purely data‐driven approach.

## 6 Limitations

This study has several limitations. For the simulations, we assumed that the noise distribution of measured *k*‐space data follows a pure Gaussian distribution. However, noise characteristics can deviate substantially from this model depending on the acquisition and reconstruction approach [45]. Additionally, linear and exponential time dependencies for metabolite dynamics were assumed. While these dependencies were selected to approximate the dynamics observed in vivo, they are unlikely to be physiologically realistic. A superior approach would involve fitting a metabolic model to in vivo data to obtain metabolic fluxes. The derived fluxes could then be used to simulate metabolite enrichment curves, better representing the dynamics in vivo [46]. Furthermore, in this study, the impact of denoising on metabolite dynamics was only evaluated qualitatively through visual inspection of metabolite concentration time courses. A more comprehensive analysis could be achieved by fitting a metabolic model to the data, enabling quantitative assessment of the impact of denoising on estimated metabolic rates [20].

To simulate a similar spatial Glx distribution to that observed in vivo, a small Glx signal was included in the CSF. Small concentrations of Glx in CSF have been reported [38]; however, it has generally been assumed that CSF does not contain MRS‐observable metabolites [47]. Exploration of the underlying reason for the observed spatial distribution of Glx labelling would be desirable.

In the lesion simulation, a full sweep of denoising performance using different truncation ranks was not performed. Quantitative analysis of the optimal rank would be an interesting direction for future work.

Rather than comparing the metabolite maps from denoised simulations directly to ground truth concentration values, we compared them to gold standard maps obtained by fitting the noiseless simulations. We chose this comparison so that the only difference between the denoised and gold standard maps was the addition of noise and subsequent denoising, minimising the impact of spectral fitting as a source of error in the comparison. However, this approach assumes that LCModel fitting works similarly well for noiseless, very noisy and denoised spectra and is not substantially biased in any of those cases.

Finally, the impact of denoising on the metabolite quantification uncertainty was limited to the variance of the error compared with the gold standard in Monte Carlo simulations. It was not assessed for in vivo data, as the uncertainty estimates of MRS quantification packages following low‐rank denoising can significantly underestimate the true uncertainty [26]. Implementation of the bootstrap method proposed by Clarke et al. to measure the uncertainty in the in vivo fitted metabolite concentration estimates following the different denoising methods would be interesting future work [26].

## 7 Conclusion

This work compared the performances of six low‐rank denoising methods on simulated and in vivo dynamic ^2^H‐MRSI data. SPIN‐SVD outperformed all other methods for denoising simulated brain ^2^H‐MRSI data and best preserved local metabolic alterations in a lesion simulation. SPIN‐SVD, GL‐HOSVD and tMPPCA all reduced noise variance for in vivo dynamic ^2^H‐MRSI data with minimal impact on the underlying Glx GM‐to‐WM contrast. Although Local PS performed better than Global PS, it did not preserve spatial metabolite variations as well as the HOSVD patch‐based methods. Of the two HOSVD‐based methods, tMPPCA was preferred due to the higher computational efficiency and objective rank selection. SPIN‐SVD and tMPPCA are both suitable denoising methods for dynamic ^2^H‐MRSI, with SPIN‐SVD better suited for clinical translation due to the simple implementation and superior preservation of metabolic alterations, while tMPPCA is superior for applications requiring absolute quantification of very low SNR metabolites.

## Author Contributions

**Anna Duguid:** conceptualisation, methodology, formal analysis, visualisation, software, writing – original draft preparation, writing – review and editing. **Fabian Niess:** conceptualisation, methodology, software, investigation, writing – review and editing. **Wolfgang Bogner:** software, funding acquisition, supervision, writing – review and editing, project administration. **Lukas Hingerl:** software, writing – review and editing. **Viola Bader:** investigation, conceptualisation, writing – review and editing. **Sabina Frese:** conceptualisation, writing – review and editing. **Aaron Osburg:** conceptualisation, writing – review and editing. **Bernard Lanz:** conceptualisation, funding acquisition, writing – review and editing. **Brayan Alves:** conceptualisation, writing – review and editing. **Cristina Cudalbu:** conceptualisation, funding acquisition, writing – review and editing. **Simon Daniel Robinson:** methodology, writing – review and editing. **Korbinian Eckstein:** methodology, writing – review and editing. **Bernhard Strasser:** conceptualisation, software, formal analysis, funding acquisition, supervision, writing – original draft preparation, writing – review and editing, project administration.

## Supporting information


**Figure S1:** Glx maps for the final repetition of (A) simulated and (B) in vivo dynamic ^2^H‐MRSI data following the four matricisation‐based denoising methods using different ranks (for all three methods the rank is user‐selected). The minimum matrix dimension for all four methods was 96; so rank = 96 is the case where no denoising is performed. Below are the corresponding plots of the ratio of GM‐to‐WM Glx signal as a function of used rank, where the ‘pure’ GM and WM signals were calculated using least squares fitting of the metabolite concentration and tissue probability maps.
**Figure S2:** Effect of the Local PS patch size on denoising performance in ^2^H‐MRSI data. (A) Local PS‐denoised ^2^H‐MRSI simulations across various patch sizes were compared with corresponding noiseless simulations to calculate Spectral RMSEs. (B) The LCModel fitted metabolite maps from the denoised simulations were compared with the gold standard maps fitted from the noiseless simulations to compute concentration RMSEs. Both spectral and concentration RMSE reduced with increasing patch size. (C) GM‐to‐WM Glx contrast and (D) weighted signal within a Lac containing lesion were both reduced as patch size increased.
**Figure S3:** Effect of the SPIN‐SVD $N_{t}$ parameter on denoising performance in ^2^H‐MRSI data. (A) Spectral RMSE and (B) concentration RMSE for denoised ^2^H‐MRSI simulations against a noiseless gold standard, where SPIN‐SVD denoising was performed with different $N_{t}$. (C) Apparent GM‐to‐WM Glx contrast plotted against $N_{t}$. (D) Metabolite maps and corresponding residual maps (difference to the gold standard) for simulations denoised using SPIN‐SVD with different $N_{t}$. Although spectral and concentration RMSE decreased with decreasing $N_{t}$, GM‐to‐WM Glx contrast was better significantly degraded for smaller $N_{t}$ values. (E) Correlation coefficient matrices for the in vivo data that has been reshaped using the SPIN‐SVD unfolding (with $N_{t}=8$) and the Stacked PS unfolding. (F) Largest 10 singular values from the SVD of the SPIN‐SVD ($N_{t}=8$) and Stacked PS matrices formed from in vivo data.
**Figure S4:** Examples of the spectral artefacts observed following denoising with SPIN‐SVD for (A) simulated and (B) in vivo ^2^H‐MRSI data. Example spectra from GM‐ and WM‐dominated voxels were selected to illustrate cases where the artefacts were particularly pronounced.
**Figure S5:** Histograms showing the voxel‐wise absolute signal errors for (A) Glc, (B) Glx and (C) HDO following different denoising methods. Absolute concentration errors were calculated voxel‐wise by subtracting the gold standard metabolite map from the denoised metabolite map. The error distributions for non‐denoised data (No LR) are displayed in grey in the background.
**Table S1:** Tissue‐specific metabolite scaling factors for dynamic ^2^H‐MRSI simulations. The repetition number (Rep) ranges from one to eight, with each repetition representing a 7‐min interval, starting 14 min after [6,6′‐^2^H_2_]‐glucose administration, as acquired in vivo. While the Glx, water and Lac signals increase linearly over time, the Glc signals follow an exponential relaxation curve. For each repetition, the tissue‐specific scaling factors were applied to each metabolite signal. The scaled metabolite signals were then multiplied with the corresponding tissue maps to generate spatiospectral data.
**Table S2:** Voxel‐wise mean of the number of signal‐components estimated using tMPPCA for simulated ^2^H‐MRSI data with different SNR and *λ*. Each tensor patch is unfolded along each index and the number of signal components estimated with MPPCA. For voxels contained within multiple patches, the number of retained components was averaged over all patches it was included in.

## Data Availability

The code used to generate the ^2^H‐MRSI simulations, as well as a MATLAB function implementing four of the denoising methods evaluated in this study (Global PS, Local PS, Stacked PS and SPIN‐SVD), is available on GitHub at: https://github.com/annaduguid/denoise‐MRSI‐tools.git. The tMPPCA and GL‐HOSVD algorithms are openly available at https://github.com/sunenj/Tensor‐MP‐PCA and https://github.com/XinyuanZhang719/gl‐hosvd.
