# Supplementary material for: Comparison of Low‐Rank Denoising Methods for Dynamic Deuterium MRSI at 7 T
Source: NMR Biomed. 2025 Aug 31;38(10):e70125. doi: 10.1002/nbm.70125 (PMC12399924; doi:10.1002/nbm.70125)
Supplement: Supplementary file 1 — Figure S1: Glx maps for the final repetition of (A) simulated and (B) in vivo dynamic 2H‐MRSI data following the four matricisation‐based denoising methods using different ranks (for all three methods the rank is user‐selected). The minimum matrix dimension for all four methods was 96; so rank = 96 is the case where no denoising is performed. Below are the corresponding plots of the ratio of GM‐to‐WM Glx signal as a function of used rank, where the ‘pure’ GM and WM signals were calculated using least squares fitting of the metabolite concentration and tissue probability maps. Figure S2: Effect of the Local PS patch size on denoising performance in 2H‐MRSI data. (A) Local PS‐denoised 2H‐MRSI simulations across various patch sizes were compared with corresponding noiseless simulations to calculate Spectral RMSEs. (B) The LCModel fitted metabolite maps from the denoised simulations were compared with the gold standard maps fitted from the noiseless simulations to compute concentration RMSEs. Both spectral and concentration RMSE reduced with increasing patch size. (C) GM‐to‐WM Glx contrast and (D) weighted signal within a Lac containing lesion were both reduced as patch size increased. Figure S3: Effect of the SPIN‐SVD Nt parameter on denoising performance in 2H‐MRSI data. (A) Spectral RMSE and (B) concentration RMSE for denoised 2H‐MRSI simulations against a noiseless gold standard, where SPIN‐SVD denoising was performed with different Nt. (C) Apparent GM‐to‐WM Glx contrast plotted against Nt. (D) Metabolite maps and corresponding residual maps (difference to the gold standard) for simulations denoised using SPIN‐SVD with different Nt. Although spectral and concentration RMSE decreased with decreasing Nt, GM‐to‐WM Glx contrast was better significantly degraded for smaller Nt values. (E) Correlation coefficient matrices for the in vivo data that has been reshaped using the SPIN‐SVD unfolding (with Nt=8) and the Stacked PS unfolding. (F) Largest 10 singular values from t [file NBM-38-e70125-s001.docx]

Supplementary material

**
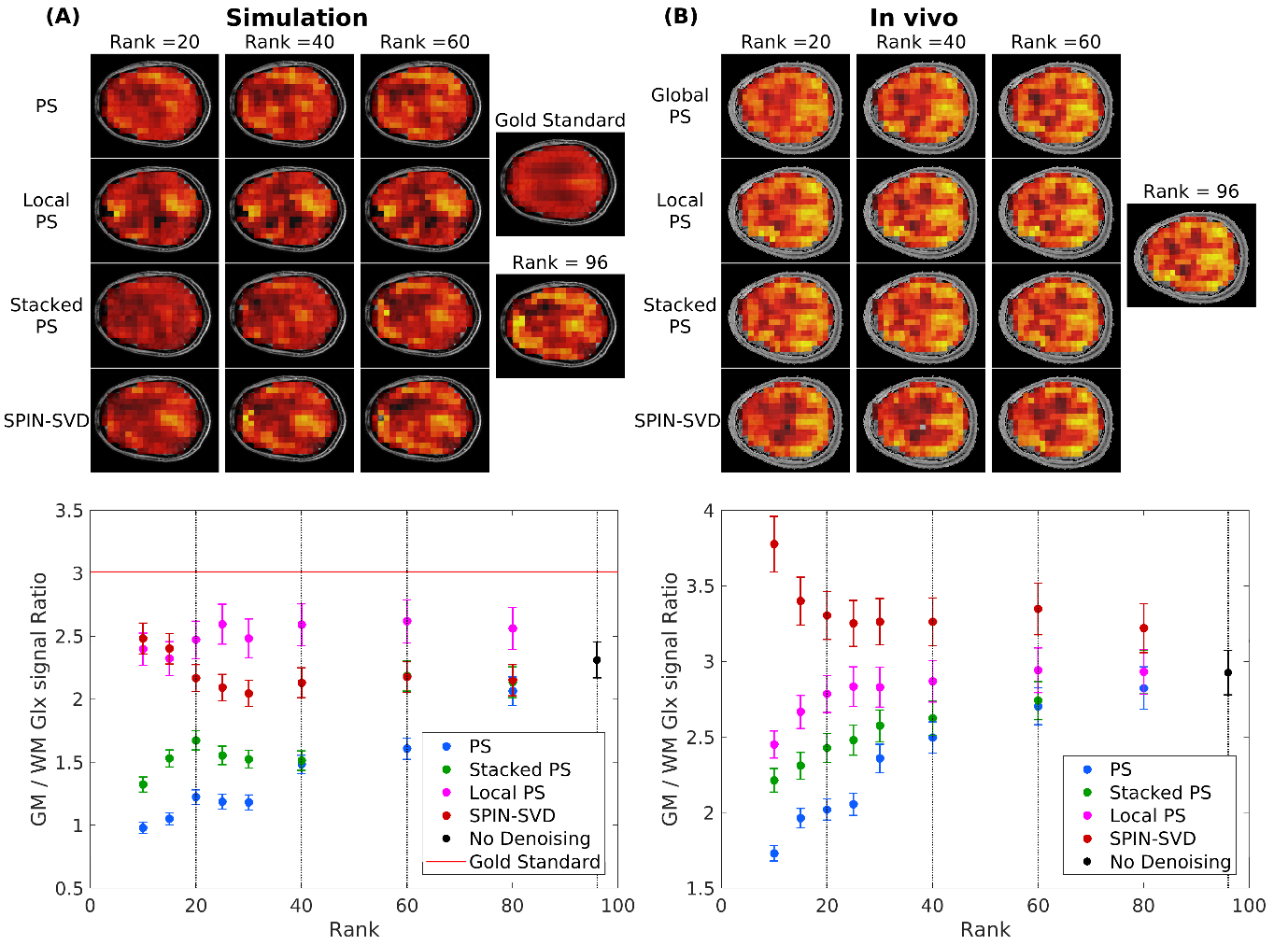
**

**Supplementary Figure 1:** Glx maps for the final repetition of (A) simulated and (B) in vivo dynamic ^2^H-MRSI data following the four matricisation-based denoising methods using different ranks (for all three methods the rank is user-selected). The minimum matrix dimension for all four methods was 96, so rank = 96 is the case where no denoising is performed. Below are the corresponding plots of the ratio of GM-to-WM Glx signal as a function of used rank, where the ‘pure’ GM and WM signals were calculated using least squares fitting of the metabolite concentration and tissue probability maps.

**
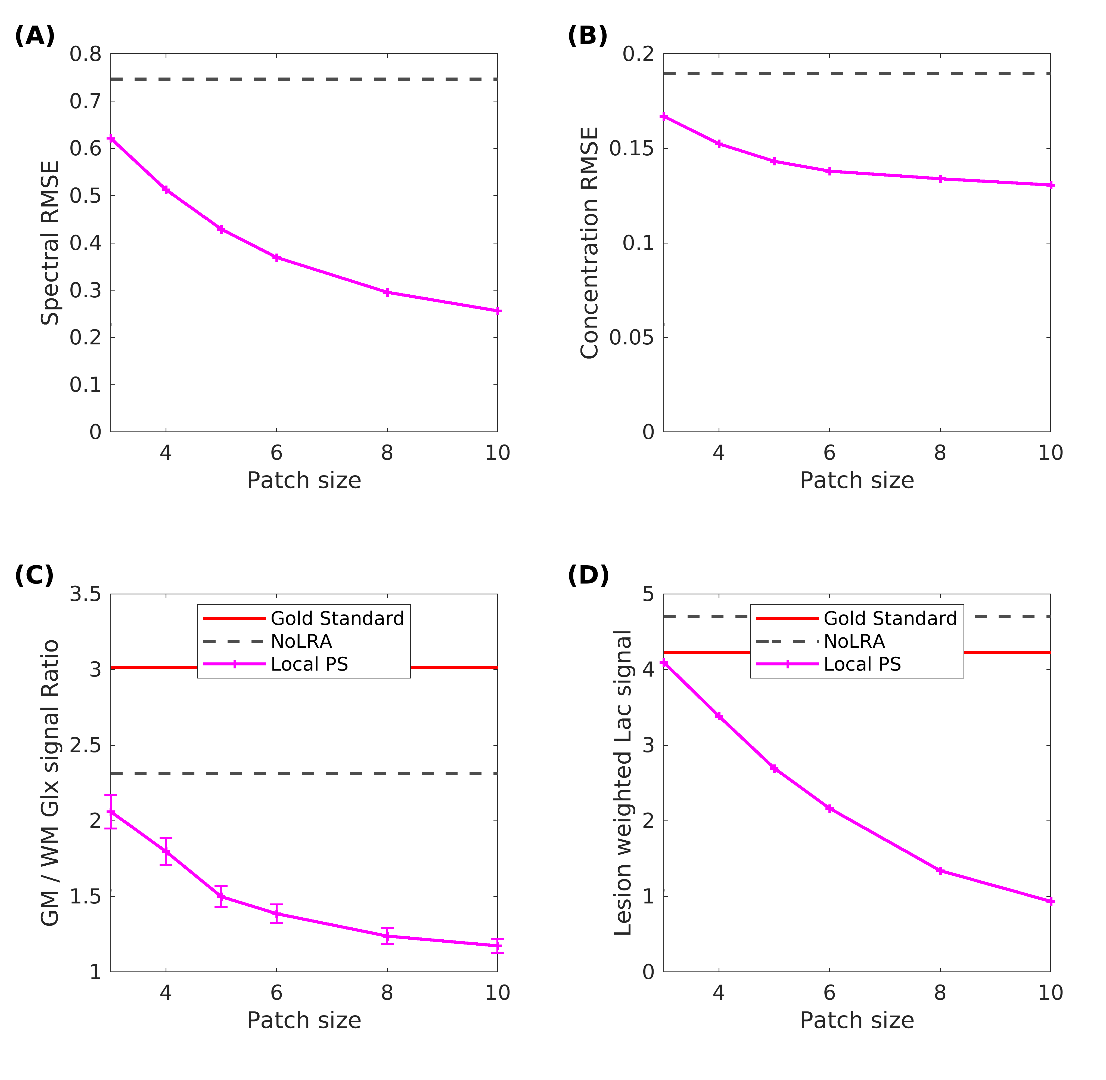
**

**Supplementary Figure 2:** Effect of the Local PS patch size on denoising performance in ^2^H-MRSI data. (A) Local PS-denoised ^2^H-MRSI simulations across various patch sizes were compared to corresponding noiseless simulations to calculate Spectral RMSEs. (B) The LCModel fitted metabolite maps from the denoised simulations were compared to the gold standard maps fitted from the noiseless simulations to compute concentration RMSEs. Both spectral and concentration RMSE reduced with increasing patch size. (C) GM-to-WM Glx contrast and (D) weighted signal within a Lac containing lesion were both reduced as patch size increased.


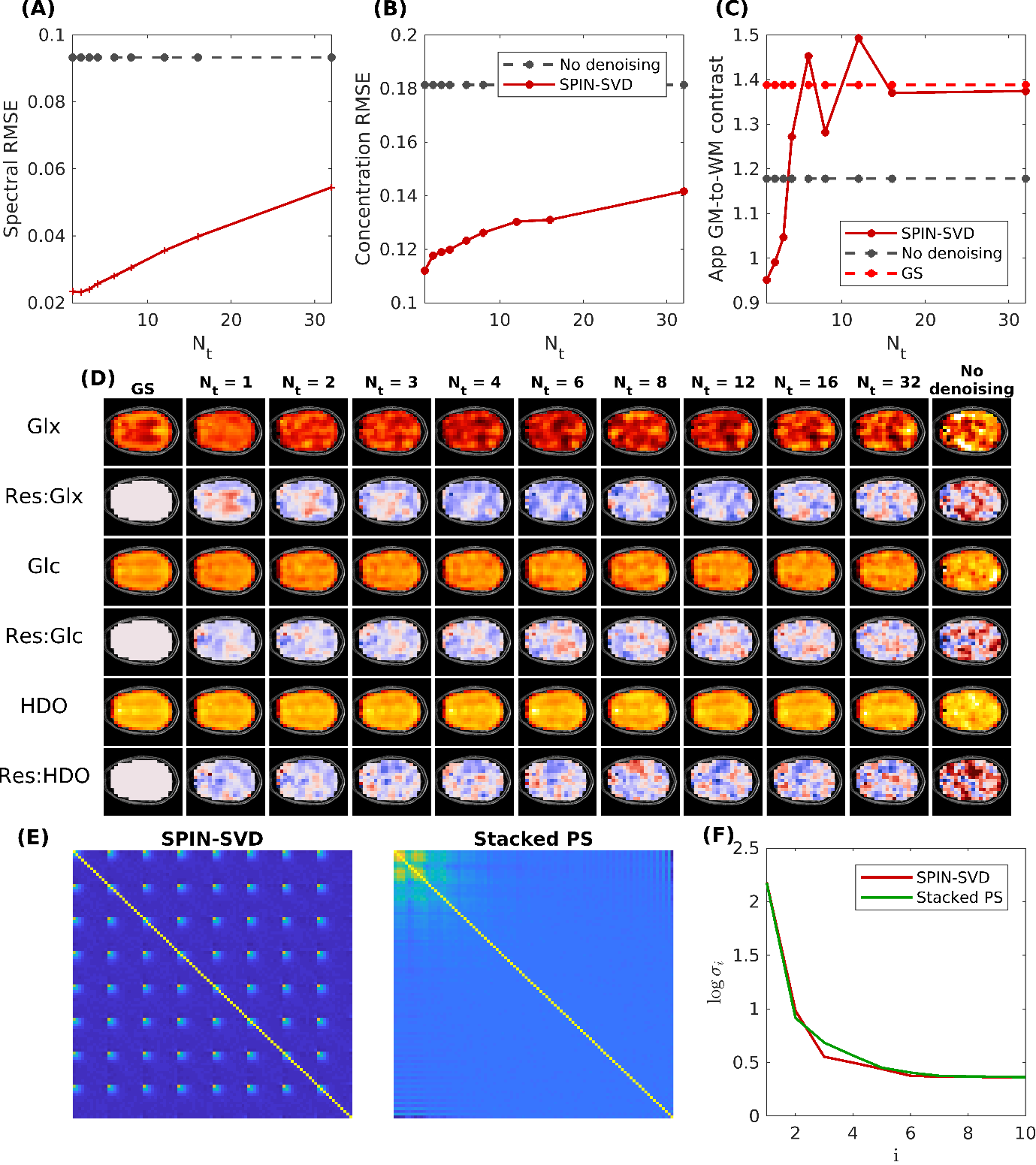


**Supplementary Figure 3:** Effect of the SPIN-SVD $N_{t}$ parameter on denoising performance in ^2^H-MRSI data. (A) Spectral RMSE and (B) concentration RMSE for denoised ^2^H-MRSI simulations against a noiseless gold standard, where SPIN-SVD denoising was performed with different $N_{t}$. (C) Apparent GM-to-WM Glx contrast plotted against $N_{t}$. (D) Metabolite maps and corresponding residual maps (difference to the gold standard) for simulations denoised using SPIN-SVD with different $N_{t}$. Although spectral and concentration RMSE decreased with decreasing $N_{t}$, GM-to-WM Glx contrast was better significantly degraded for smaller $N_{t}$ values. (E) Correlation coefficient matrices for the in vivo data that has been reshaped using the SPIN-SVD unfolding (with $N_{t}=8$) and the Stacked PS unfolding. (F) Largest 10 singular values from the SVD of the SPIN-SVD ($N_{t}=8$) and Stacked PS matrices formed from in vivo data.

**
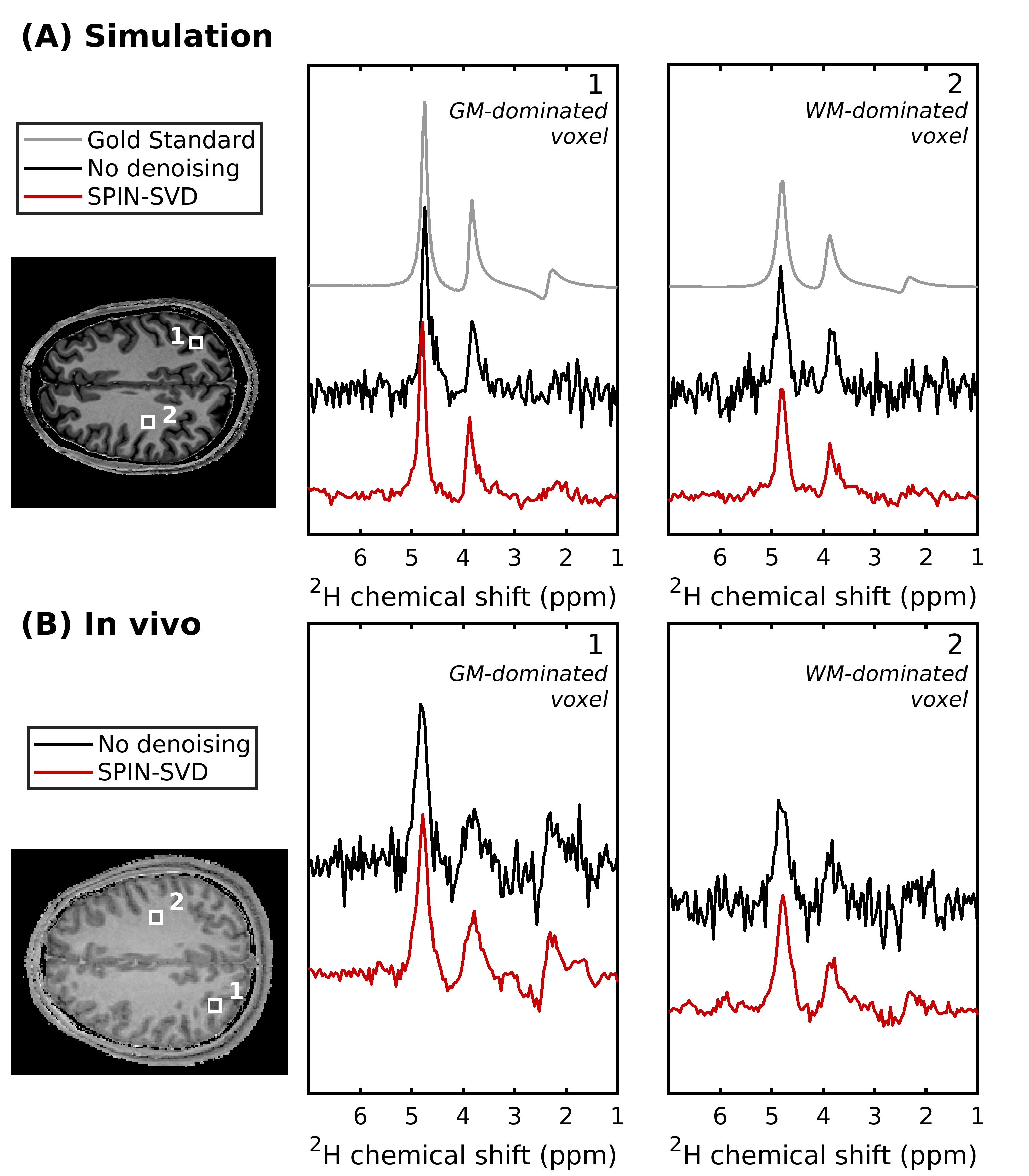
**

**Supplementary Figure 4:** Examples of the spectral artefacts observed following denoising with SPIN-SVD for (A) simulated and (B) in vivo ^2^H-MRSI data. Example spectra from GM- and WM-dominated voxels were selected to illustrate cases where the artefacts were particularly pronounced.


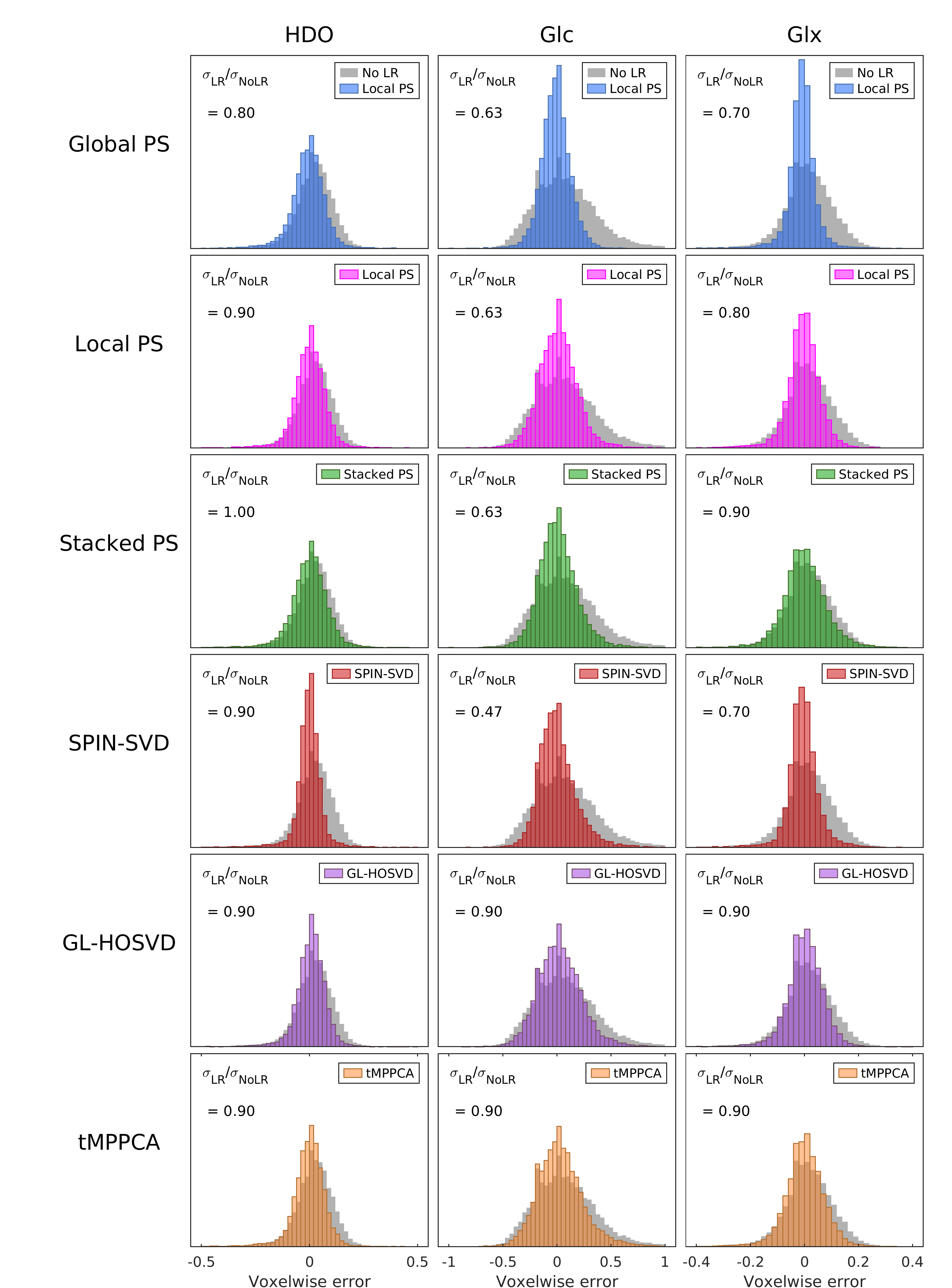


**Supplementary Figure 5:** Histograms showing the voxel-wise absolute signal errors for (A) Glc, (B) Glx, and (C) HDO following different denoising methods. Absolute concentration errors were calculated voxel-wise by subtracting the gold standard metabolite map from the denoised metabolite map. The error distributions for non-denoised data (No LR) are displayed in grey in the background.

| Tissue Type | METABOLITE | | | |
| --- | --- | --- | --- | --- |
|  | *Glc* | *Glx* | *water* | *Lac* |
| GM | 0.7*(1.04-exp(-0.35*Rep)) | 1*(0.143+0.107*Rep) | 2.5*(0.886+0.014*Rep) | - |
| WM | 0.7*(1.04-exp(-0.35*Rep)) | 0.25*(-0.0714+0.171*Rep) | 2*(0.886+0.014*Rep) | - |
| CSF | 1*(1.04-exp(-0.35*Rep)) | 0.125*(0.143+0.107*Rep) | 3*(0.886+0.014*Rep) | - |
| Lesion | 1*(1.04-exp(-0.35*Rep)) | 0.1*(0.143+0.107*Rep) | 2.5*(0.886+0.014*Rep) | 1*(0.143 + 0.107*Rep) |

**Supplementary Table 1:** Tissue-specific metabolite scaling factors for dynamic ^2^H-MRSI simulations. The repetition number (Rep) ranges from one to eight, with each repetition representing a 7-minute interval, starting 14 minutes after [6,6’-^2^H_2_]-glucose administration, as acquired in vivo. While the Glx, water and Lac signals increase linearly over time, the Glc signals follow an exponential relaxation curve. For each repetition, the tissue-specific scaling factors were applied to each metabolite signal. The scaled metabolite signals were then multiplied with the corresponding tissue maps to generate spatiospectral data.

|  |  | FLATTENING DIMENSION | | | | |
| --- | --- | --- | --- | --- | --- | --- |
|  |  | *t* | *x* | *y* | *z* | *T* |
| SNR | 5 | 44.07 ± 0.87 | 3.00 ± 0.00 | 3.06 ± 0.05 | 4.00 ± 0.00 | 8.00 ± 0.00 |
|  | 9 | 44.03 ±0.73 | 3.00 ± 0.00 | 3.15 ± 0.10 | 4.00 ± 0.00 | 8.00 ± 0.00 |
|  | 15 | 44.94 ± 0.84 | 3.00 ± 0.00 | 3.58 ± 0.21 | 4.00 ± 0.00 | 8.00 ± 0.00 |
|  | 20 | 45.07 ± 0.65 | 3.00 ± 0.00 | 3.76 ± 0.20 | 4.00 ± 0.00 | 8.00 ± 0.00 |
|  | 25 | 45.25 ± 0.79 | 3.03 ± 0.04 | 3.86 ± 0.15 | 4.00 ± 0.00 | 8.00 ± 0.00 |
|  | 30 | 45.60 ± 0.87 | 3.18 ± 0.14 | 3.87 ± 0.14 | 4.00 ± 0.00 | 8.00 ± 0.00 |
| λ | 0.0 | 44.06 ± 0.64 | 3.00 ± 0.00 | 3.09 ± 0.09 | 4.00 ± 0.00 | 8.00 ± 0.00 |
|  | 0.5 | 43.93 ± 0.58 | 3.00 ± 0.00 | 3.10 ± 0.08 | 4.00 ± 0.00 | 8.00 ± 0.00 |
|  | 1.0 | 44.03 ± 0.73 | 3.00 ± 0.00 | 3.15 ± 0.10 | 4.00 ± 0.00 | 8.00 ± 0.00 |
|  | 1.5 | 44.80 ± 0.79 | 3.00 ± 0.00 | 3.22 ± 0.14 | 4.00 ± 0.00 | 8.00 ± 0.00 |
|  | 2.0 | 44.60 ± 0.57 | 3.00 ± 0.00 | 3.20 ± 0.13 | 4.00 ± 0.00 | 8.00 ± 0.00 |
| In vivo | | 40.34 ± 0.59 | 3.00 ± 0.00 | 4.00 ± 0.00 | 4.00 ± 0.00 | 8.00 ± 0.00 |

**Supplementary Table 2:** Voxel-wise mean of the number of signal-components estimated using tMPPCA for simulated ^2^H-MRSI data with different SNR and λ. Each tensor patch is unfolded along each index and the number of signal components estimated with MPPCA. For voxels contained within multiple patches, the number of retained components was averaged over all patches it was included in.
